# Multimodal stimulation screens reveal unique and shared genes limiting T cell fitness

**DOI:** 10.1016/j.ccell.2024.02.016

**Published:** 2024-04-08

**Authors:** Chun-Pu Lin, Pierre L. Levy, Astrid Alflen, Georgi Apriamashvili, Maarten A. Ligtenberg, David W. Vredevoogd, Onno B. Bleijerveld, Ferhat Alkan, Yuval Malka, Liesbeth Hoekman, Ettai Markovits, Austin George, Joleen J.H. Traets, Oscar Krijgsman, Alex van Vliet, Joanna Poźniak, Carlos Ariel Pulido-Vicuña, Beaunelle de Bruijn, Susan E. van Hal-van Veen, Julia Boshuizen, Pim W. van der Helm, Judit Díaz-Gómez, Hamdy Warda, Leonie M. Behrens, Paula Mardesic, Bilal Dehni, Nils L. Visser, Jean-Christophe Marine, Gal Markel, William J. Faller, Maarten Altelaar, Reuven Agami, Michal J. Besser, Daniel S. Peeper

**Affiliations:** 1 Division of Molecular Oncology and Immunology, Oncode Institute, The Netherlands Cancer Institute, Plesmanlaan 121, 1066 CX Amsterdam, the Netherlands; 2 Proteomics Facility, The Netherlands Cancer Institute, Plesmanlaan 121, 1066 CX Amsterdam, the Netherlands; 3 Division of Tumor Biology and Immunology, The Netherlands Cancer Institute, Plesmanlaan 121, 1066 CX Amsterdam, the Netherlands; 4 Division of Oncogenomics, The Netherlands Cancer Institute, Plesmanlaan 121, 1066 CX Amsterdam, the Netherlands; 5 Department of Pathology, VU University Amsterdam, 1081 HV Amsterdam, the Netherlands; 6 Biomolecular Mass Spectrometry and Proteomics, Center for Biomolecular Research and Utrecht Institute for Pharmaceutical Sciences, Utrecht University, Padualaan 8, 3584 CH Utrecht, the Netherlands; 7 Tumor Immunology and Immunotherapy Group, Vall d'Hebron Institute of Oncology (VHIO), Vall d'Hebron Barcelona Hospital Campus, 08035 Barcelona, Spain; 8 Department of Hematology and Medical Oncology, University Medical Center, Johannes Gutenberg-University, 55131 Mainz, Germany; 9 Research Center for Immunotherapy (FZI), University Medical Center, Johannes Gutenberg-University, 55131 Mainz, Germany; 10 Ella Lemelbaum Institute for Immuno-oncology and Melanoma, Sheba Medical Center, Ramat Gan 52612, Israel; 11 Department of Clinical Microbiology and Immunology, Faculty of Medicine, Tel Aviv University, Tel-Aviv 6997801, Israel; 12 Davidoff Cancer Center and Samueli Integrative Cancer Pioneering Institute, Rabin Medical Center, Petach Tikva 4941492, Israel; 13 Felsenstein Medical Research Center, Faculty of Medicine, Tel Aviv University, Tel Aviv 6997801, Israel; 14 Laboratory for Molecular Cancer Biology, VIB Center for Cancer Biology, 3000 Leuven, Belgium; 15 Laboratory for Molecular Cancer Biology, Department of Oncology, KU Leuven, 3000 Leuven, Belgium

**Keywords:** T cells, dysfunction, activation-induced cell death, exhaustion, cancer immunotherapy, *Dap5*, *Icam1*, *Ctbp1*, CRISPR-Cas9 screen, effector function

## Abstract

Genes limiting T cell antitumor activity may serve as therapeutic targets. It has not been systematically studied whether there are regulators that uniquely or broadly contribute to T cell fitness. We perform genome-scale CRISPR-Cas9 knockout screens in primary CD8 T cells to uncover genes negatively impacting fitness upon three modes of stimulation: (1) intense, triggering activation-induced cell death (AICD); (2) acute, triggering expansion; (3) chronic, causing dysfunction. Besides established regulators, we uncover genes controlling T cell fitness either specifically or commonly upon differential stimulation. *Dap5* ablation, ranking highly in all three screens, increases translation while enhancing tumor killing. Loss of *Icam1*-mediated homotypic T cell clustering amplifies cell expansion and effector functions after both acute and intense stimulation. Lastly, *Ctbp1* inactivation induces functional T cell persistence exclusively upon chronic stimulation. Our results functionally annotate fitness regulators based on their unique or shared contribution to traits limiting T cell antitumor activity.

## Highlights

• Multimodal screens identify T cell fitness regulators impeding antitumor activity
• *Dap5* ablation increases translation enhancing T cell fitness upon various stimulations
• Perturbing ICAM1-LFA1-mediated T cell clustering amplifies T cell effector functions
• *Ctbp1* inactivation strengthens T cell persistence exclusively upon chronic stimulation

## Graphical abstract

**Figure:**
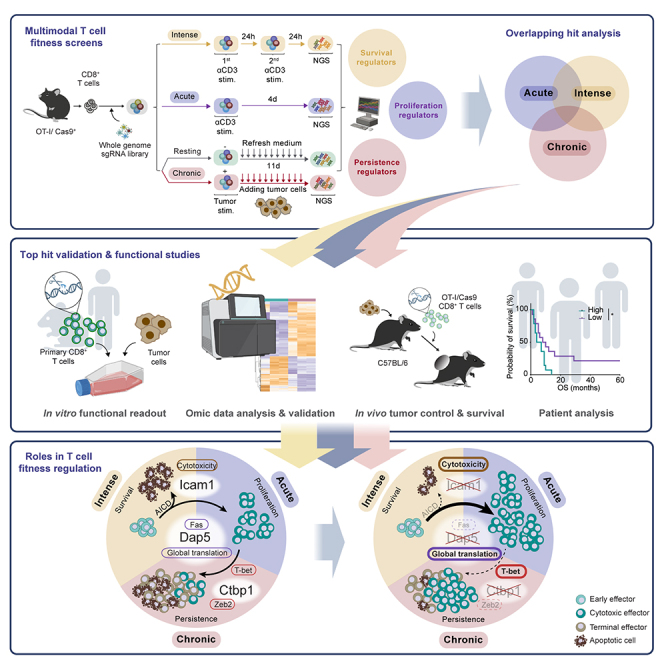

## Teaser

Lin et al. perform multimodal genome-wide CRISPR knockout screens in primary CD8 T cells for genes controlling fitness upon differential stimulation. They identify *Dap5*, *Icam1*, and *Ctbp1*, which are functionally annotated and characterized based on their unique or shared contribution to traits limiting T cell antitumor activity.

## Introduction

Immune checkpoint blockade (ICB) and adoptive cell transfer (ACT) have become promising therapies for many cancer types.^1^^,^^2^^,^^3^^,^^4^^,^^5^^,^^6^ However, their benefit is limited by lack of therapy response or resistance, manifesting at several stages.^7^^,^^8^^,^^9^ Tumor-intrinsic resistance mechanisms are pleiotropic, as revealed by genomic studies^10^^,^^11^^,^^12^^,^^13^^,^^14^ and CRISPR-based screens.^14^^,^^15^^,^^16^^,^^17^^,^^18^^,^^19^^,^^20^^,^^21^^,^^22^ Immunotherapy responses strongly depend on tumor-reactive effector T cells.^23^^,^^24^ Encountering tumor-antigens triggers T cell activation and expansion while stimulating the production of cytotoxic molecules.^25^^,^^26^ However, the tumor microenvironment (TME) can limit T cell antitumor efficacy by impacting various fitness traits, including survival, proliferation, and functional persistence.^27^^,^^28^^,^^29^ This can be driven by distinct stimulation contexts, resulting in cell differentiation into several states. For example, different intensity and duration of stimulation determine whether, and to what extent, T cells can exert their effector functions.^30^^,^^31^^,^^32^

Tumor-specific T cells face repetitive antigenic stimulation in the TME, which can be intense and trigger activation-induced cell death (AICD). Survival under AICD is one of the first mechanisms that determine cell number shortly after T cell receptor (TCR) stimulation.^33^^,^^34^^,^^35^ This apoptotic cell death is enabled by upregulating death receptors after activation, most prominently Fas.^36^^,^^37^^,^^38^ Although AICD has been observed mostly in a viral infection context, there is accumulating evidence that it limits T cell antitumor activity^39^^,^^40^^,^^41^ and chimeric antigen receptor (CAR) T cell therapy benefit.^42^^,^^43^^,^^44^ Therefore, blocking AICD may improve T cell survival in tumors.^45^

Proliferative capacity is a second main fitness trait for antigen-specific effector T cells to expand and eliminate tumor cells,^46^ serving as a key indicator of immunotherapy response.^47^^,^^48^^,^^49^ Hence, harnessing cell proliferation represents an opportunity to obtain sufficient tumor-reactive T cells.

Chronic antigen stimulation accounts for a third signal hampering T cell fitness, limiting “effector-persistence” or dysfunction. It is a process in which cells gradually lose their cytotoxicity and proliferation capacity, eventually becoming unresponsive to tumor-antigen stimulation.^50^^,^^51^^,^^52^^,^^53^^,^^54^^,^^55^ This is associated with the induction of inhibitory receptors such as PD-1, LAG-3,^56^^,^^57^^,^^58^^,^^59^^,^^60^ and activity of transcription factors like T-BET, EOMES, IRF4, NFAT, TCF-1, and TOX.^61^^,^^62^^,^^63^^,^^64^^,^^65^^,^^66^^,^^67^^,^^68^^,^^69^^,^^70^^,^^71^ These factors establish a transcriptionally and epigenetically distinct cell state,^67^^,^^72^^,^^73^^,^^74^^,^^75^ associated with terminal differentiation and reduced effector-persistence.^76^^,^^77^^,^^78^ Targeting inhibitory receptors reinvigorates effector function, improving clinical benefit.^79^^,^^80^^,^^81^ Thus, interfering with genes limiting effector persistence under chronic stimulation may allow for more durable immunotherapy responses.

The multifactorial causes of T cells losing their fitness upon differential stimulation are the subject of intense study, as they impede tumor control. While several players were identified previously by CRISPR screening,^82^^,^^83^^,^^84^^,^^85^^,^^86^^,^^87^^,^^88^^,^^89^^,^^90^^,^^91^^,^^92^^,^^93^^,^^94^^,^^95^^,^^96^^,^^97^^,^^98^^,^^99^^,^^100^^,^^101^^,^^102^^,^^103^^,^^104^^,^^105^^,^^106^ it has not yet been systematically addressed whether critical factors control only specific aspects or simultaneously regulate multiple T cell fitness features. This is what we set out to study here, in an unbiased, genome-wide fashion, investigating three different stimulation modalities, namely: intense, acute, and chronic stimulation.

## Results

### Multimodal function-based genome-wide CRISPR knockout screens for genes contributing to T cell fitness upon differential stimulation

We set out to recapitulate the aforementioned three key processes determining T cell antitumor activity. First, in immunocompetent mice bearing ovalbumin (OVA)-expressing melanomas, we identified endogenous OVA-specific CD8 tumor-infiltrating lymphocytes (TILs) showing reduced viability compared to non-specific T cells (Figure S1A), in agreement with AICD by intense antigen stimulation. Second, in an ACT mouse model, we observed increased proliferation of transferred OVA-specific T cells in tumors compared to spleens 3 days after ACT (Figure S1B), indicating rapid proliferation upon antigen stimulation. Third, transferred T cells expressed higher levels of inhibitory receptors in tumors compared to spleens at tumor endpoint, showing exhaustion induced by chronic antigen stimulation (Figure S1C).

**Figure 1:**
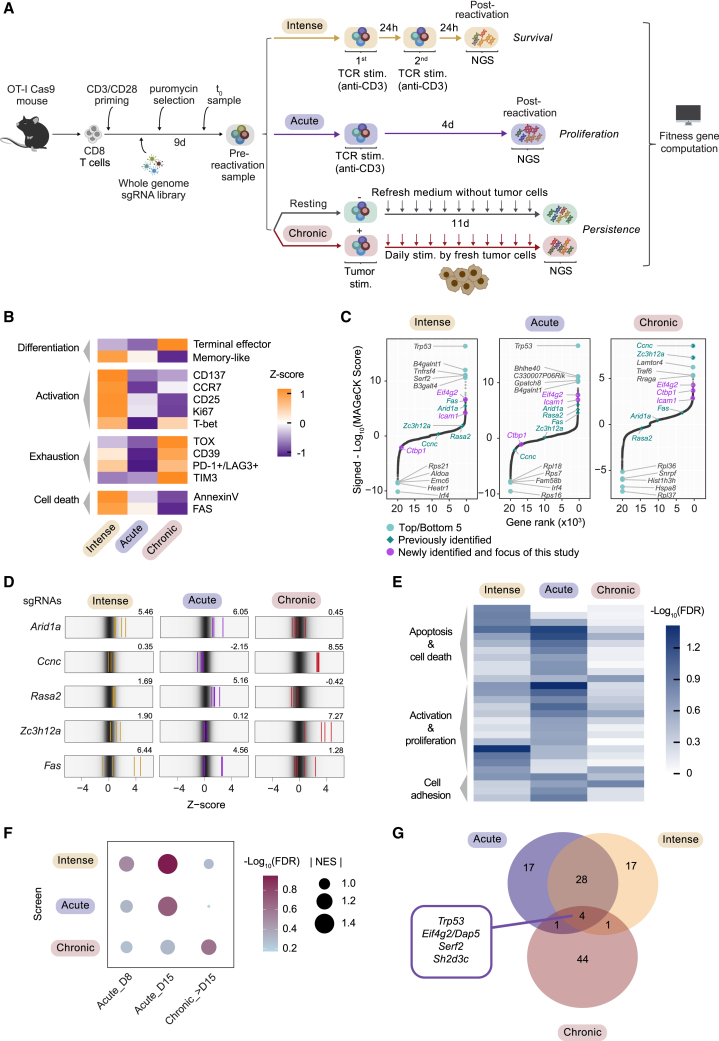
Multimodal function-based genome-wide CRISPR knockout screens for genes contributing to T cell fitness upon differential stimulation (A) T cell stimulation screens setup. (B) Marker expression heatmap from flow cytometry analysis of T cells stimulated with indicated conditions as in (A). *Z* score indicates the fold change to resting cells. (C) MAGeCK analysis of screen results (Table S1). (D) Enrichment of individual sgRNAs targeting genes identified from published T cell screens. Numbers above plots indicate signed -Log_10_(MAGeCK score). (E) GSEA of GO biological process from screen hits (Table S1). FDR: false discovery rate. (F) GSEA of CD8 lineage gene sets^107^ from screen hits (Tables S1 and S5). NES: normalized effect size. (G) Numbers of overlapping genes from top 50 hits of each screen. Genes are listed by average effect size (Table S1).

These results prompted us to use unbiased CRISPR-Cas9 knockout screens to uncover genes in primary murine CD8 T cells that contribute to fitness loss upon differential stimulation. The TME comprises several factors influencing T cell responses. To identify players regulating specific effector traits under defined stimulation contexts, to avoid confounders, and to ensure robust library coverages, we designed three independent genome-scale CRISPR-Cas9 knockout screens: intense (two successive 24 h-TCR stimulations, enriching for sgRNAs promoting survival), acute (single 24 h-TCR stimulation followed by 4 days proliferation, enriching for sgRNAs promoting proliferation), and chronic (repetitive tumor-antigen stimulation for 11 days, enriching for sgRNAs promoting persistence) (Figure 1A).

We crossed Cas9-GFP^108^ mice with OT-I mice^109^ and isolated naive OT-I/Cas9 cells. After 48 h-priming with anti-CD3 and anti-CD28, a genome-wide sgRNA library^110^ was retrovirally transduced. Cells were pharmacologically selected for 6 days. A t_0_ library reference sample was harvested 24 h post-selection and a pre-reactivation sample (primed effector cells) was taken right before the start of all screens to confirm dropout of essential-gene-targeting sgRNAs.^111^ (Figures S1D and S1E; Table S1).

For the intense stimulation/survival screen, library-containing cells (referring to CD8 effector cells, unless otherwise specified) were challenged twice with 24 h-anti-CD3 stimulation, causing cell viability to drop progressively, indicating strong survival pressure (Figure S1F). For the acute stimulation/proliferation screen, one-time 24 h-anti-CD3 stimulation was applied. Stimulated cells were cultured for additional 3 days, allowing cell expansion (Figure S1G). For the chronic stimulation/persistence screen, T cells were continuously stimulated by D4M.OVA mouse melanoma cells^112^ for 11 days at a fixed T cell:tumor cell ratio. A resting group was included where medium was refreshed without tumor cells, avoiding screening for proliferation regulators independent of chronic stimulation. As expected, 11 days chronic stimulation triggered the upregulation of multiple exhaustion markers (Figure S1H),^56^^,^^57^^,^^58^^,^^113^^,^^114^ while inducing apoptosis^115^ (Figure S1I) and terminal differentiation^116^ (Figure S1J). Furthermore, these cells exhibited reduced cytotoxicity compared to resting cells upon restimulation (Figures S1K and S1L), adopting a dysfunction phenotype. Systematic flow cytometry analysis of cells under all screen conditions confirmed and extended the previously described phenotypes (Figures 1B and S1M). Although we cannot exclude confounding signals contributing to the final population in each setting, the characterization of our screen settings supports their key phenotypes (survival, proliferation, and persistence) serving as discriminating factors.

Cells were collected at each screen endpoint, genomic DNA was isolated, and sgRNAs were PCR-amplified and sequenced. sgRNA enrichment from output samples was compared to either pre-reactivation (intense and acute screens) or resting (chronic screen) samples by MAGeCK analysis^117^ (Figures 1C; Table S1). We identified several regulators discovered previously, including *Arid1a*, *Rasa2*, *Ccnc*, and *Zc3h12a* (alias *Regnase-1*^118^).^90^^,^^92^^,^^102^^,^^104^ Moreover, sgRNAs targeting *Fas*, a key positive regulator of cell death and apoptosis, were enriched particularly in the intense stimulation screen^34^^,^^36^ (Figures 1C and 1D), all illustrating the screen robustness. Gene ontology (GO) term^119^^,^^120^ gene set enrichment analysis (GSEA)^121^^,^^122^ with MAGeCK-ranked hits showed enrichment of expected biological processes related to apoptosis and proliferation for the intense and acute stimulation screens, respectively, and to activation for both (Figures 1E; Table S1). As the GO term database lacks exhaustion signatures, we derived gene sets from published single-cell RNA-seq (scRNA-seq) data^107^ (Table S5). Highlighting the relevance of the chronic stimulation screen, the exhaustion signature (Chronic_>D15) was enriched exclusively in the chronic setting (Figures 1F; Tables S1 and S5). Integrating the top 50 enriched genes from all screens, we identified four shared hits: *Trp53*, *Eif4g2* (alias *Dap5*^123^), *Serf2*, and *Sh2d3c*, whose depletion positively influenced T cell fitness upon all three stimulation modes (Figures 1G; Table S1).

### *Dap5* inactivation alleviates global inhibition of effector T cell fitness and enhances tumor-killing capacity

**Figure 2:**
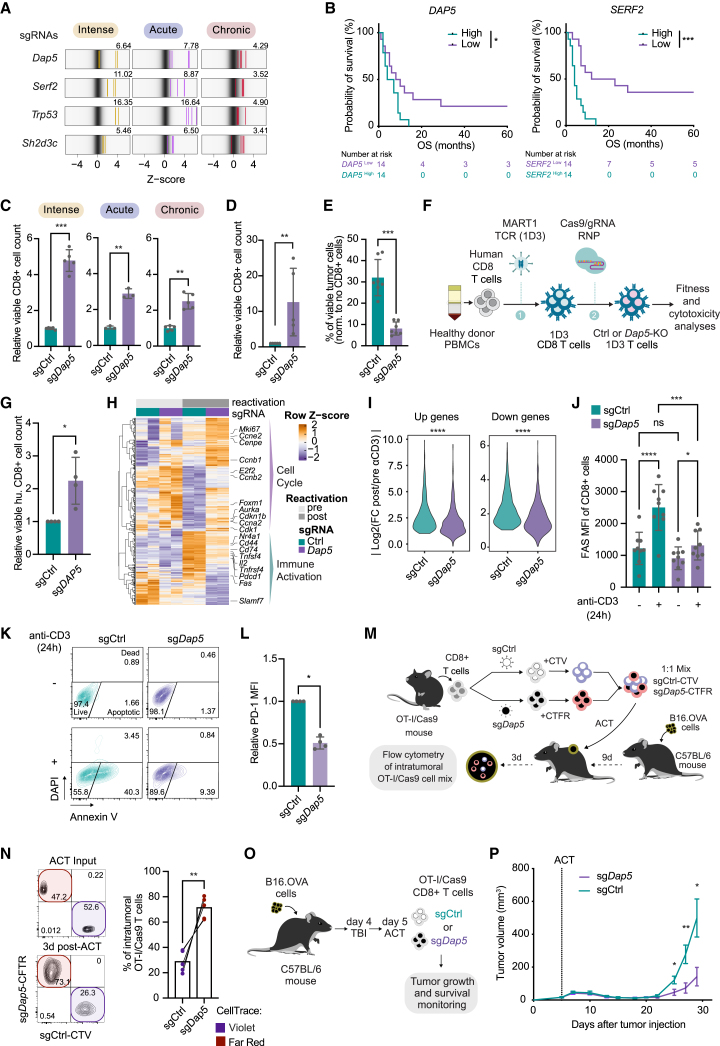
*Dap5* inactivation alleviates global inhibition of effector T cell fitness and enhances tumor-killing capacity (A) Enrichment of individual sgRNAs targeting overlapping genes (4 sgRNAs/gene). Numbers above plots indicate signed -Log_10_(MAGeCK score). (B) Kaplan-Meier OS curves of patients receiving TIL therapy (Besser cohort)^124^^,^^125^ with top and bottom third highest and lowest (33.3%) *DAP5* or *SERF2* expression in TIL products. Significance calculated by regular log rank test. (C) Viable cell number under indicated stimulation conditions, analyzed with two-tailed paired t test (n = 3–5 biological replicates). (D) Viable cell count after 96 h co-culturing with D4M.OVA cells, analyzed with Mann-Whitney test (n = 5 biological replicates). (E) Tumor cell survival after co-culture with Ctrl and *Dap5*-KO T cells, analyzed with two-tailed paired t test (n = 7 biological replicates). (F) Outline for generating human CD8 cells expressing MART-1-reactive 1D3 TCR. 1. Retroviral-transduction 2. Nucleofection. RNPs: ribonucleoprotein particles. (G) Viable human Ctrl and *DAP5*-KO MART-1 CD8 cell count after 72 h co-culture with A375-HLA-A^∗^02:01/MART-1 cells, analyzed with Mann–Whitney test (n = 4 biological replicates). (H) Transcriptomic profiling heatmap of indicated T cells pre/post 24 h-CD3 stimulation, showing significantly (p value <0.001) differentially expressed genes (Table S2). (I) Absolute Log_2_(fold-change) of CD3 stimulation-induced upregulated and downregulated genes, related to (H) (Table S2). (J) Flow cytometry analysis on cells, with or without 24 h-CD3 stimulation, analyzed with one-way ANOVA, followed by a Tukey post-hoc test (n = 9 biological replicates). (K) Representative flow cytometry plots (n = 2 biological replicates) showing apoptotic T cells. (L) Flow cytometry analysis of T cells 4 days after CD3 stimulation, analyzed with Mann-Whitney test (n = 4 biological replicates). (M) Outline of *in vivo* competition assay. (N) Left: Flow cytometry plot showing T cell mixes, input or isolated from tumors 3 days after ACT. Right: Quantification of *in vivo* competition assay, analyzed with two-tailed paired t test (n = 5 mice/group). (O) Outline of ACT tumor model. (P) B16.OVA tumor growth in mice treated with either Ctrl or *Dap5*-KO T cells, as in (O), analyzed with a two-tailed unpaired t test per time point. Error bars represent SEM (n = 9 mice/group). Error bars indicate SD, unless otherwise specified. ^∗^p < 0.05; ^∗∗^p < 0.01; ^∗∗∗^p < 0.001; ^∗∗∗∗^p < 0.0001.

To assess clinical relevance of overlapping hits—*Trp53*, *Dap5*, *Serf2*, and *Sh2d3c*—(Figure 2A) from all three screens, we queried a cohort of patients with melanoma receiving TIL therapy,^124^^,^^125^ where RNA-seq was performed for TIL products prior to infusion (Besser M.J., RNA-seq data unpublished). We stratified patients receiving TIL with the highest and lowest expression of the indicated genes. Patients receiving TILs expressing low *DAP5* or *SERF2* showed significantly longer overall survival (OS) (Figures 2B; Table S2). No significant effect was seen in TILs with low *SH2D3C* or *TP53* expression (Figure S2A; Table S2). Querying the role of *DAP5* and *SERF2* in cell exhaustion, we extended our analysis to 49 publicly available scRNA-seq data from pan-cancer cohorts from the TISCH database.^126^ Expression of both genes was significantly higher in the exhausted CD8 subset than in conventional CD8 cells (Figure S2B; Table S2). Thus, we identified two fitness genes, *DAP5* and *SERF2*, whose ablation enhances T cell fitness under all three stimulation types, and whose expression levels correlate with exhaustion and unfavorable TIL response.

To validate *Dap5*, OT-I/Cas9 cells were retrovirally transduced with sgRNA targeting *Dap5*. *Dap5*-KO T cells showed significantly higher cell count under all stimulation settings (Figure 2C), including chronic CD3 stimulation (Figure S2C), confirming that *Dap5* inactivation improves general T cell fitness. It was important to determine whether *Dap5*-KO impacts effector functions. We co-cultured *Dap5*-KO or Ctrl T cells with different murine melanoma cell lines expressing TCR-matched antigens (OT-I:OVA^109^ or Pmel:gp100^127^^,^^128^). In line with our screen result, *Dap5-*KO T cell number increased when cultured with tumor cells (Figures 2D and S2D), accompanied by a superior tumor cell elimination (Figures 2E and S2E). This result demonstrates an enhanced effector function upon *Dap5* ablation, which is not restricted to TCR-antigen specificity (Figure S2E). To extend these observations, we inactivated *DAP5* in human CD8 cells expressing MART-1 TCR^129^ for functional assessment (Figure 2F). We observed that its inactivation in human T cells also resulted in increased cell numbers upon CD3 stimulation (Figure S2F), and in co-culture with MART-1 tumor cells (Figure 2G). These data indicate that *Dap5* inactivation enhances T cell fitness and efficacy under various stimulation conditions.

DAP5 is a close homolog of the translation initiation factor EIF4G1, influencing both cap-dependent and cap-independent mRNA translation.^130^^,^^131^^,^^132^^,^^133^^,^^134^^,^^135^ To understand its role in regulating translation in T cells, we first assessed the effect of *Dap5* ablation on global translation. We incubated Ctrl and *Dap5*-KO T cells with methionine analog L-homopropargylglycine (HPG) that is incorporated into newly synthesized proteins, to determine overall translation rate. *Dap5*-KO increased global translation (Figure S2G). Consistently, a strongly reduced level of the translation repressor 4E-BP1^136^ was observed (Figure S2H), together suggesting that DAP5 may act as a translational inhibitor in T cells.

To dissect whether this increase in translation is driven by a subset of highly translated mRNAs or rather a global effect, we performed polysome and total mRNA sequencing. A comparison between the polysome and translational profiling revealed similar overall translation efficiency between Ctrl and *Dap5*-KO cells (Figure S2I; Table S2), suggesting a global increase in translation. We compared the transcriptomic profiles of *Dap5*-KO and Ctrl T cells with or without CD3 stimulation, showing increased expression of cell cycle genes alongside a moderate immune activation program (Figures 2H; Table S2). Furthermore, *Dap5*-KO cells had dampened global transcriptional changes upon stimulation (Figures 2I; Table S2), accompanied by a significantly lower FAS expression (Figure 2J). By combining DAPI and annexin V staining, we observed a significant reduction of apoptosis in *Dap5*-KO cells (Figures 2K and S2J), accompanied by a slight increase in viability after TCR stimulation (Figure S2K). Antibody-mediated blockade of FasL benefited sgCtrl-expressing cells upon stimulation while having a minor effect on *Dap5-*deficient cells, supporting the notion that Fas signaling is diminished in *Dap5-*KO cells, thereby aiding their survival (Figure S2L). *Dap5* ablation also resulted in 2-fold downregulation of PD-1 (Figure 2L), but did not affect IFNγ production upon stimulation (Figure S2M).

These results prompted us to investigate whether *Dap5* inactivation benefits T cell function *in vivo*, where multiple challenges must be overcome for effective tumor control. We first examined whether *Dap5* ablation increases T cell numbers within the tumor in an *in vivo* competition experiment (Figure 2M). In line with our *in vitro* data, *Dap5*-KO cells were more abundant in tumors compared to Ctrl cells (Figures 2N and S2N), with increased proliferative activity (Figure S2O). To assess the clinical potential of *Dap5* inactivation, we carried out an ACT therapy in a B16.OVA melanoma mouse model. Mice underwent total body irradiation (TBI) as a lymphodepleting regimen before receiving either Ctrl or *Dap5-*KO T cells,^137^ and tumor growth and survival were monitored (Figure 2O). Mice that received *Dap5*-KO cells showed improved tumor control (Figures 2P and S2P) and survival (Figure S2Q). Our observations both *in vitro* and *in vivo* demonstrate that *Dap5*-KO in effector cells enhances global translation while suppressing FAS expression, together contributing to improving fitness upon stimulation, boosting their antitumor efficacy.

### Loss of *Icam1*-mediated homotypic T cell interactions amplifies CD8 T cell expansion and improves effector functions shortly after TCR stimulation

**Figure 3:**
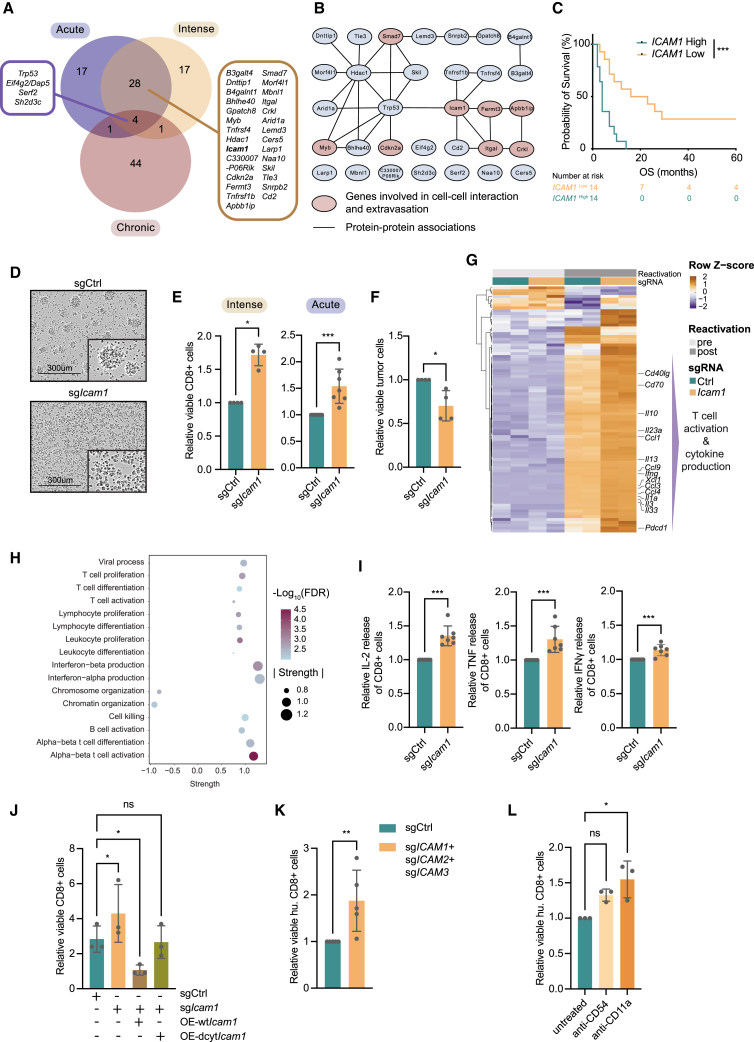
Loss of *Icam1*-mediated homotypic T cell interactions amplifies CD8 T cell expansion and improves effector functions shortly after TCR stimulation (A) Overlapping genes from top 50 hits from each screen, genes are ranked by average effect size. (B) STRING protein-protein interaction analysis of shared targets from the two boxes in (A) (32 genes). Interactions include direct (physical) and indirect (functional) associations. (C) Kaplan-Meier OS curves of patients receiving TIL therapy^124^^,^^125^ (Besser cohort) with high or low *ICAM1* expression in TIL products. Significance calculated with regular log rank test. (D) Microscopy images of indicated T cells 24 h after CD3 stimulation (n = 7 biological replicates). (E) Viable Ctrl and *Icam1*-KO T cell counts under indicated stimulation conditions as in the screens, analyzed with Mann-Whitney test (n = 4–7 biological replicates). (F) Viable B16.OVA cells after 4 days co-culture with indicated T cells, analyzed with Mann-Whitney test (n = 4 biological replicates). (G) Transcriptomic profiling heatmap of indicated T cells with or without 24 h-CD3 stimulation, showing significantly (p value <0.001) differentially expressed genes (Table S3). (H) Proteomic STRING enrichment analysis of differentially expressed proteins comparing *Icam1*-KO with Ctrl T cells after 24 h-CD3 stimulation, showing top enriched GO biological process (ranked by enrichment strength (Log_10_(observed/expected)) with FDR < 0.1 (Table S3). (I) Flow cytometry-based cytokine bead array showing cytokines released in the culture medium of indicated T cells after 24 h-CD3 stimulation, analyzed with Mann-Whitney test (n = 7 biological replicates). (J) Viable cell counts of Ctrl or *Icam1*-KO T cells ectopically expressing wild type (wt) or mutated *ICAM1* (lacking the intracellular domain, dcyt). Cell count was assessed 4 days after CD3 stimulation; analyzed with one-way ANOVA with Holm-Sidak’s multiple comparisons test (n = 3 biological replicates). OE: overexpression. (K) Viable cell counts of Ctrl or *ICAM1/2/3*-KO human T cells 1 week after 24 h-CD3 stimulation, analyzed with Mann-Whitney test (n = 5 biological replicates). (L) Viable cell counts of human CD8 cells 1week after CD3 stimulation with or without CD54 (ICAM1) or CD11a (LFA1) blocking antibodies, analyzed with Kruskal-Wallis test with Dunn’s post-hoc test (n = 3 biological replicates). Error bars indicate SD. ^∗^p < 0.05; ^∗∗^p < 0.01; ^∗∗∗^p < 0.001; ^∗∗∗∗^p < 0.0001.

Next, we characterized overlapping hits from the acute and intense stimulation screens (Figure 3A). Their ablation rendered T cells more resistant to AICD while boosting proliferation upon stimulation, producing a sufficient cell pool facilitating tumor control. For prioritization, we performed STRING analysis^138^ with all genes in this category (n = 32). This identified multiple genes involved in cell-cell interaction or extravasation pathways (Figures 3B and S3A; Table S3), suggesting a crucial role of cell-cell interaction in regulating T cell fitness upon TCR stimulation. Among candidates involved in cell-cell interaction regulation, integrin subunit alpha l (*Itgal*), encoding the integrin alpha L chain that represents one-half of the lymphocyte function-associated antigen 1 (LFA1) heterodimer,^139^ and its ligand ICAM1^140^ showed up as top enriched hits. Although the crucial role of LFA1 expression on T cell migration is well established,^141^ ICAM1 expression is mostly studied in endothelial and tumor cells, where it serves as a crucial binding partner for LFA1 on T cells^142^; its role in CD8 cells is less clear.^143^^,^^144^^,^^145^^,^^146^^,^^147^^,^^148^ Patients receiving TILs with lower *ICAM1* expression showed significantly longer OS (Figures 3C; Table S2), implying its clinical relevance in cancer immunotherapy.

To validate the role of homotypic T cell interaction regulated by *Icam1* upon TCR stimulation, *Icam1* was ablated from T cells and stimulated with CD3 antibody for 24 h. Immediately after, as reported, Ctrl cells formed dense, homotypic cell-cell aggregates^147^ but not *Icam1*-KO cells (Figure 3D). After both intense and acute stimulation, *Icam1*-KO T cells showed higher viable cell counts (Figure 3E). More importantly, their tumor cell-killing ability was significantly enhanced (Figure 3F), indicating a positive impact of *Icam1* inactivation on T cell effector function.

To dissect the underlying mechanism, we performed transcriptomic analysis on *Icam1*-KO T cells upon TCR stimulation. Prior to stimulation, no difference was observed with Ctrl cells. However, upon stimulation, gene transcripts involved in effector function were markedly increased in *Icam1-*KO cells, indicating a stronger effector phenotype (Figures 3G; Table S3). Simultaneous proteomic analyses showed a strong enrichment of processes involved in T cell activation in *Icam1*-KO cells (Figure 3H; Table S3), in line with the transcriptomic profiles. Additional flow cytometry analysis revealed enhanced cytokine production following TCR stimulation by *Icam1*-KO (Figure 3I). Notably, *Icam1-*KO cells did not show higher PD-1/LAG3 co-expression compared to control cells 7 days post-stimulation (Figure S3B). Furthermore, higher viable cell counts were accompanied by an increased KLRG1-/CD62L+ population of *Icam1*-KO cells 7 days after stimulation (Figure S3C), suggesting a higher potential for memory precursor development.^149^^,^^150^

To understand whether the induced effector function triggered by *Icam1*-KO is achieved by targeting cell-cell interaction, we studied the different roles of the extracellular and intracellular domains of ICAM1 in T cells. We re-expressed either wild-type *Icam1* (wt*Icam1*) or a *Icam1* mutant lacking its intracellular domain (dcyt*Icam1*) in *Icam1*-KO cells. After stimulation, dcyt*Icam1* cells exhibited a similar clustering phenotype as Ctrl cells (Figure S3D), indicating that the intracellular domain of ICAM1 does not contribute to cell-cell interaction. To determine whether the extracellular domain alone is sufficient for reversing the positive effects of *Icam1* loss, we assessed viable cell counts 4 days after CD3 stimulation. Of note, wt*Icam1* cells showed higher ICAM1 expression levels than parental cells (Figure S3E), which led to T cell hyperclustering, even prior to stimulation (Figure S3D). This also led to impaired cell viability (Figure 3J) and a reduced memory precursor population (Figure S3F). These results reveal that the clustering behavior of T cells correlates with their cell viability, effector function, and phenotype.

To translate these findings to a more clinically relevant setting, it is noteworthy that humans express five ICAM family members (*ICAM1-5*). Only *ICAM1*-*3* are expressed on lymphocytes, with dominant roles of ICAM1 and ICAM3 binding to LFA1.^151^^,^^152^^,^^153^^,^^154^ In contrast, murine CD8 cells express almost exclusively *Icam1*, and the *Icam3* gene was inactivated in mice during evolution (Figure S3G).^155^ Therefore, we generated single, double, and triple KOs in human CD8 T cells. 1 week after CD3 stimulation, the double (*ICAM1* and *ICAM3*) and triple (*ICAM1*, *ICAM2*, and *ICAM3*) knockouts resulted in significant increases in viable cell counts (Figures 3K and S3H).

Based on our data with human T cells, we reasoned that targeting LFA1 by a blocking antibody (CD11a) may prevent clustering, which is likely more efficient than blocking ICAM1 (CD54) alone. Indeed, treatment of human primary CD8 cells with CD11a antibody resulted in better expansion, outperforming CD54 antibody treatment (Figure 3L). Thus, preventing homotypic T cell interactions by targeting the ICAM-LFA1 axis recapitulates the improved effector phenotype achieved by genetic manipulation, which may merit exploration for cell therapy.

### *Ctbp1* ablation induces T cell persistence exclusively under chronic stimulation, associated with reduced ZEB2/T-bet-dependent terminal differentiation

**Figure 4:**
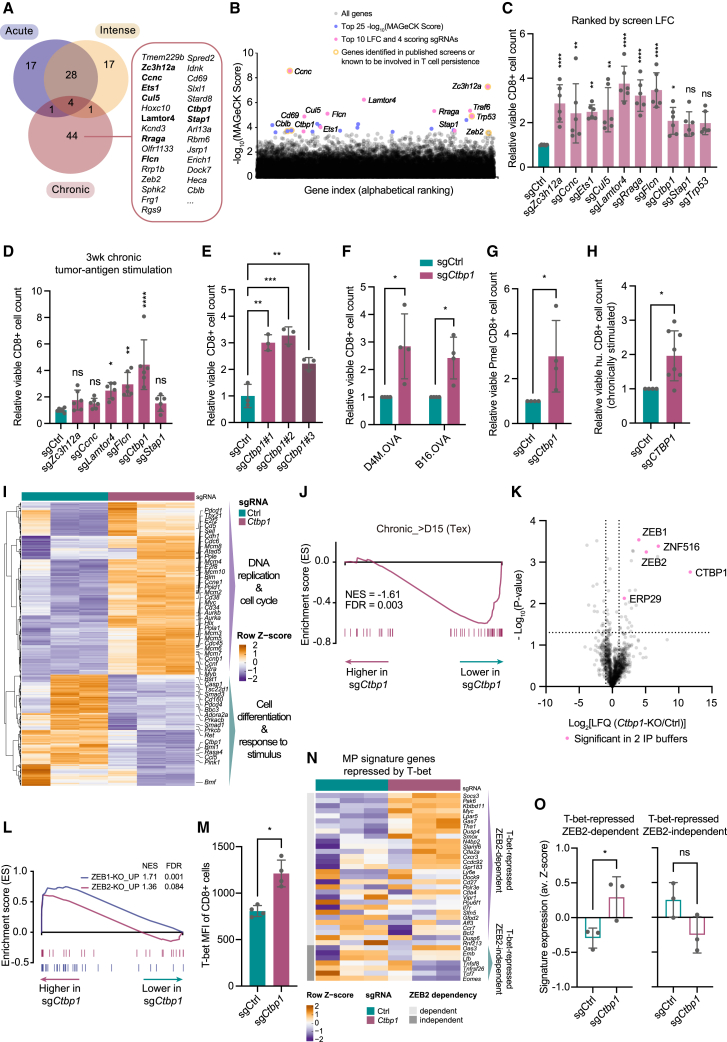
*Ctbp1* ablation induces T cell persistence exclusively under chronic stimulation, associated with reduced ZEB2/T-bet-dependent terminal differentiation (A) Overlapping genes from top 50 hits of each screen, listing top exclusive genes from the chronic stimulation screen (ranked by effect size). Genes selected for validation are in bold. (B) -Log_10_(MAGeCK score) for all genes in the chronic stimulation screen. (C) *In vitro* validation of top-ranking hits exclusively from the chronic stimulation screen, showing relative viable T cell count after 11 days chronic D4M.OVA stimulation as in the screen. Top 25 genes with 4/4 enriched sgRNAs were re-ranked by effect size (LFC, log_2_(fold change)), and top 10 genes were selected for validation. Cell count fold-change was normalized to resting condition (Figure S4B). Analyzed with one-way ANOVA, followed by a Dunnett post-hoc test from three biological replicates with two different sgRNAs per replicate (n = 3x2). (D) Relative viable cell counts of T cells expressing indicated sgRNAs after prolonged (3 weeks) chronic D4M.OVA stimulation. Genes with significantly increased cell count after chronic stimulation (Figure 4C), but without proliferation (dis-) advantage (+/− 25% change) under resting condition (Figure S4B), were selected for prolonged chronic *in vitro* stimulation. Analyzed with one-way ANOVA, followed by a Dunnett post-hoc test from three biological replicates with two different sgRNAs per replicate (n = 3x2). (E) Relative viable cell counts of T cells expressing Ctrl or different sgRNAs targeting *Ctbp1* after > 2 weeks chronic tumor-antigen-stimulation. Analyzed with one-way ANOVA with Holm-Sidak’s multiple comparisons test (n = 3 biological replicates). (F) Relative viable cell counts of Ctrl and *Ctbp1*-KO T cells after > 2 weeks chronic B16.OVA or D4M.OVA stimulation, analyzed with Mann-Whitney test (n = 4 biological replicates). (G) Relative viable cell counts of indicated Pmel/Cas9 T cells after >2 weeks chronic B16 tumor cell stimulation. Analyzed with Mann-Whitney test (n = 4 biological replicates). (H) Relative viable cell counts of human Ctrl and *Ctbp1*-KO MART-1 CD8 cells after 3–5 weeks co-culturing with D10 melanoma cells (expressing endogenous MART-1 antigen). Analyzed with Mann-Whitney test from four biological replicates with two different sgRNAs per replicate (n = 4x2). (I) Transcriptomic profiling heatmap of indicated T cells 3 weeks post D4M.OVA chronic stimulation, showing significantly (p value <0.001) differentially expressed genes. Pure T cells were sorted by flow cytometry (Table S4). (J) GSEA of exhaustion signature, Chronic_>D15 (UP in Tex, as in Figure 1F), comparing *Ctbp1*-KO to Ctrl T cells after chronic stimulation. (K) Immunoprecipitation mass spectrometry (IP-MS) analysis of CTBP1 from wt OT-I/Cas9 cells after CD3 stimulation (n = 2 independent experiments with different IP buffers, see also Figure S4H; Table S4). Proteins identified from both independent IP-MS are in pink. 1% Triton X-100 IP buffer was used. (L) GSEA of ZEB1-KO_UP (AIGNER_ZEB1_TARGETS)^156^ and ZEB2-KO_UP^157^ signatures (UP in *ZEB1* or *ZEB2* KO cells), comparing Ctrl and *Ctbp1*-KO T cells after chronic stimulation (Table S5). (M) Flow cytometry analysis of indicated T cells after 3 weeks chronic stimulation, analyzed with two-tailed paired t test (n = 4 biological replicates). (N) Expression of memory precursor (MP) signature genes known to be repressed by T-bet but either dependent or independent of ZEB2 regulation,^158^ related to I) (Table S4). (O) Quantification of (N). Analyzed with two-tailed unpaired t test (n = 3 biological replicates). Error bars indicate SD. ^∗^p < 0.05; ^∗∗^p < 0.01; ^∗∗∗^p < 0.001; ^∗∗∗∗^p < 0.0001.

Lastly, we wished to characterize genes that, instead, uniquely contribute to only a single T cell fitness setting: chronic stimulation. From the top hits exclusively identified in the chronic stimulation screen (Figure 4A), multiple genes have been reported previously, either with potential for cancer immunotherapy (*Regnase-1*,^102^ *Cblb*,^159^ and *Ccnc*^92^) or associated with exhaustion (*Cd69*)^160^ or terminal differentiation (*Zeb2*).^158^ This encouraged us to focus on this group to identify targets that upon inactivation could prolong tumor-specific T cell persistence and sustain effector function.

We took the top 25 genes from MAGeCK analysis, from which we prioritized the validation of the 10 genes showing the largest log_2_ fold-change (LFC) and scoring with 4/4 sgRNAs in the library (sgRNAs ranking below the alpha cutoff in the MAGeCK analysis)^117^ (Figures 4B and S4A). *Regnase-1*-KO cells were strongly enriched, corroborating the screen system.^102^ *Trp53* was included as a reference gene whose inactivation induces cell proliferation independent of chronic antigen stimulation.^161^^,^^162^^,^^163^ sgRNAs perturbing the top 10 genes were transduced into OT-I/Cas9 cells. Cells were then either chronically stimulated by adding fresh D4M.OVA cells at a fixed T cell:tumor cell ratio or refreshed without adding tumor cells (“resting”) for 11 days, identical to the screen setting (Figure 1A). 8/10 of the hits were validated by increased viable cell counts (Figure 4C). From the parallel analysis in the resting condition, depletion of four hits (including *Tp53*) resulted in either increased or decreased viable cell counts in the absence of chronic tumor-antigen stimulation (Figure S4B), which were excluded from further analysis.

For prioritization, T cells ablated for top validated hits (stimulation-dependent) were exposed to longer and stronger chronic tumor-antigen stimulation for 3 weeks, causing clear exhaustion (Figures S4C–S4E). *Ctbp1*-KO cells had acquired the most pronounced increase in cell count (Figure 4D). This effect was confirmed with additional sgRNAs (Figure 4E), chronic CD3 stimulation (Figure S4F), across tumor types (Figure 4F), and a different matching antigen-TCR pair (Figure 4G). Corroborating these results in human CD8 cells, we found that *CTBP1* ablation again caused a stronger persistence phenotype after chronic stimulation with antigen-matched tumor cells (Figures 4H and S4G).

To understand the role of *CTBP1* in T cell persistence, we performed transcriptomic profiling after 3 weeks of chronic stimulation. Many DNA replication and cell cycle regulating genes were upregulated in *Ctbp1*-KO T cells, whereas genes involved in cell differentiation and responses were downregulated (Figures 4I; Table S4). In line with our chronic stimulation screen results, we found that an exhaustion signature (Figure 1F)^107^ was negatively enriched in *Ctbp1*-KO cells (Figure 4J), implying that *Ctbp1* inactivation slows down cell exhaustion upon chronic stimulation.

Since CTBP1 functions as a transcriptional corepressor,^164^ we set out to identify potential interactors in T cells upon stimulation. We performed immunoprecipitation mass spectrometry (IP-MS) with buffers differing in stringency, identifying several potential CTBP1 interactors. As expected, ZEB1 and ZEB2, previously established CTBP1 interactors,^165^^,^^166^ showed up as top hits (Figures 4K and S4H; Table S4). They are reciprocally expressed during CD8 T cell development;^167^ *Zeb2* is crucial in promoting terminal effector differentiation, whereas *Zeb1* is required for maintaining the homeostasis of memory cells.^157^^,^^158^^,^^168^^,^^169^ Together with our IP-MS data, these results suggest an important role for CTBP1 in regulating ZEB1/ZEB2-mediated T cell differentiation. To investigate whether ZEB1 and ZEB2 control the phenotype of *Ctbp1*-KO T cells, we performed GSEA analysis. An enrichment of both Zeb1-knockout (AIGNER_ZEB1_TARGETS)^156^ and *Zeb2*-knockout signatures^157^ was found (Figures 4L; Table S5). Moreover, *Ctbp1-KO* T cells adopted a less terminal differentiated effector phenotype, as measured by gene sets from two independent studies on effector differentiation in either a chronic^73^ (Figure S4I; Table S4) or acute^170^ (Figure S4J; Table S4) LCMV infection mouse model. These data suggest that CTBP1 cooperates with ZEB2 to regulate effector terminal differentiation.

Next, we investigated how CTBP1 affects terminal differentiation and effector status of CD8 T cells. We observed that T-box transcription factor *Tbx21 (T-bet*), a key regulator of antigen-induced effector function,^171^^,^^172^^,^^173^ was one of the top upregulated genes in the transcriptomic analysis (Figure S4K). This was confirmed in *Ctbp1*-KO cells after chronic stimulation (Figure 4M). *T-bet*, together with ZEB2, drives terminal differentiation by promoting terminally differentiated effector (TE) genes while repressing memory precursor (MP) genes.^158^ Using previously reported signatures,^158^ we found that 3 weeks after chronic tumor-antigen stimulation, multiple T-bet-repressed/ZEB2-dependent MP genes (Figures 4N and 4O; Table S4), as well as T-bet-induced/ZEB2-independent TE genes (Figures S4L and S4M; Table S4), were significantly higher expressed in *Ctbp1*-KO cells. These results indicate a collaborative role of CTBP1 together with T-bet and ZEB2 in regulating T cell differentiation: *Ctbp1*-KO induces T-bet expression, thereby enhancing effector function. In contrast, *Ctbp1*-KO restrains ZEB2’s inhibitory function on MP genes to promote effector terminal differentiation. Thus, we hypothesize that *Ctbp1*-KO in matured effector cells endows them with a hybrid phenotype with enhanced effector function but delayed terminal differentiation, prolonging functional-effector persistence.

### Blocking CTBP1-mediated terminal T cell differentiation preserves T cell effector function and enables long-term tumor control

**Figure 5:**
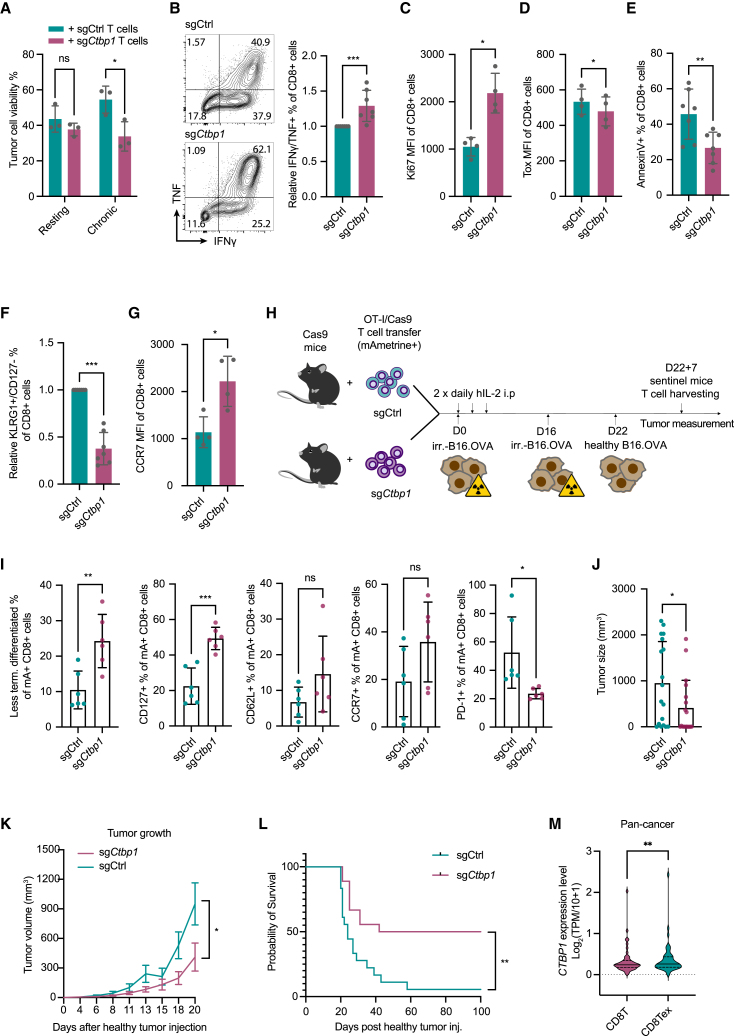
Blocking CTBP1-mediated terminal T cell differentiation preserves T cell effector function and enables long-term tumor control (A) Crystal violet (CV) staining quantification of viable B16.OVA tumor cells after 4 days co-cultured with equal amounts of indicated OT-I/Cas9 cells that were rested or chronically stimulated with tumor cells for 3 weeks. Analyzed with two-tailed paired t test (n = 3 biological replicates). (B) Flow cytometry analysis of IFNγ and TNF double-positive population of indicated OT-I/Cas9 cells after 3 weeks D4M.OVA stimulation. Cells were re-stimulated with PMA/Ionomycin prior to analysis. Left: representative plot. Right: Quantification. Analyzed with Mann-Whitney test (n = 7 biological replicates). (C) As in (B), showing Ki67 expression, analyzed with two-tailed paired t test (n = 4 biological replicates). (D–G) Flow cytometry analyses of indicated marker expression on Ctrl and *Ctbp1*-KO T cells after 3 weeks chronic D4M.OVA stimulation, analyzed with two-tailed paired t test (D, E, G) or Mann-Whitney test (F). Data points indicate biological replicates. (H) Outline of *in vivo* prolonged tumor antigen stimulation ACT experiment, related to Figures 5I–5L, S5H, and S5I. (I) Flow cytometry analyses of marker expression on transferred T cells isolated from tumors 7 days after viable tumor cell transplantation, as in (H). KLRG1-/CD127+ cells are considered less terminally differentiated. Analyzed with two-tailed unpaired t test (n = 6 mice/group). (J) Tumor size 20 days after viable tumor injection, as in (H) (when first mouse dropped out at tumor endpoint), showing data pooled from 2 independent experiments. Analyzed with two-tailed unpaired t test. (n = 18 mice/group). (K) Measurement of tumor outgrowth as in (H), analyzed with two-tailed unpaired t test (n = 18 mice/group). Error bars indicate SEM. (L) Kaplan-Meier plot depicting the survival of B16.OVA tumor-bearing mice as in (H), analyzed with regular log rank test (n = 18 mice/group). (M) *CTBP1* expression in CD8Tex and CD8T cells from 49 scRNA-seq datasets (pan-cancer). Expression level was directly derived from TISCH2 website analysis^126^ (Table S2). TPM: transcripts per million. Analyzed with Wilcoxon test (n = 49 independent datasets). Error bars indicate SD unless otherwise specified. ^∗^p < 0.05; ^∗∗^p < 0.01; ^∗∗∗^p < 0.001; ^∗∗∗∗^p < 0.0001.

To examine the functionality of *Ctbp1*-KO T cells, and to test our hypothesis, we determined their tumor-killing capacity and effector phenotype after 3 weeks chronic tumor-antigen stimulation *in vitro*. *Ctbp1*-KO T cells showed improved tumor-eliminating capacity post-chronic stimulation, but not in a resting condition (Figures 5A and S5A), consistent with the chronic stimulation screen and extending the prioritization results (Figure S4B). This was paralleled by enhanced cytokine production (Figures 5B and S5B), stronger cell proliferation capacity upon restimulation (Figure 5C) and upregulation of IL-2 receptor (Figure S5C). Moreover, although more activated, *Ctbp1*-KO cells did not show a pronounced exhaustion phenotype (Figures 5D and S5D), and they were more resistant to cell apoptosis (Figure 5E). On the other hand, several terminal differentiation markers were lower expressed (Figures 5F and S5E),^64^^,^^116^ whereas central memory markers^174^^,^^175^^,^^176^^,^^177^^,^^178^^,^^179^^,^^180^ were upregulated (Figure 5G, S5F and S5G) in *Ctbp1*-KO cells after chronic stimulation. These data support our hypothesis that depleting *Ctbp1* in effector T cells causes them to retain a hyperactivated yet less terminal differentiated status, indicating the potential of creating long-lasting effectors.

This prompted us to set up an *in vivo* model to study prolonged tumor-antigen stimulation in immune-competent mice. As the classic ACT tumor model can be influenced by a short-term proliferation advantage, we developed a prolonged tumor-antigen-stimulation ACT model. Transferred T cells were challenged *in vivo* by multiple rounds of irradiated-tumor cell injection prior to viable tumor cell transplantation, extending the chronic stimulation duration (Figure 5H). This allowed us to focus on the long-term persistence of transferred T cells, minimizing the proliferation confounder during early expansion. To avoid possible rejection of OT-I/Cas9 cells, Cas9-expressing recipient mice (C57BL/6J background) were used. 7 days post viable tumor cell injection, transferred T cells were harvested from tumors and lymph nodes. *Ctbp1*-KO cells isolated from tumors, but not from lymph nodes, showed an increased population of less terminally differentiated cells, displaying central memory marker expression (Figures 5I and S5H), in line with our observation *in vitro*. Furthermore, mice receiving *Ctbp1*-KO T cells showed better tumor control 20 days after viable tumor cell injection (44 days post ACT) (Figures 5J and 5K). We observed 9/18 complete responses for ACT with *Ctbp1*-KO cells compared to 1/18 for control cells (Figure S5I), resulting in significantly longer overall tumor-free survival (Figure 5L).

Lastly, to investigate a potential role of CTBP1 in regulating CD8 cells in cancer immunotherapy, we analyzed scRNA-seq data from TILs of patients with melanoma treated with ICB.^181^ We found a significant correlation between low *CTBP1* expression in CD8 TILs and favorable ICB response (Figure S5J). Similarly, we observed a trend of better survival when patients received TIL expressing low levels of *CTBP1* (Figure S5K; Table S2, Besser TILs cohort).^124^^,^^125^ In line with our findings for *DAP5* and *SERF2*, *CTBP1* expression was significantly higher in the exhausted CD8 subset than in conventional CD8 cells in pan-cancer cohorts from the TISCH database^126^ (Figures 5M; Table S2). Both published patient data and our own *in vivo* data support our finding that *Ctbp1* inactivation in effector T cells reinforces their effector function, delaying cells from terminal differentiation and exhaustion. The enhanced effector persistence allows for improved tumor control and prolonged survival in a chronic tumor-antigen-stimulation mouse model, meriting therapeutic exploration of *Ctbp1* inactivation for T cell therapy.

## Discussion

**Figure 6:**
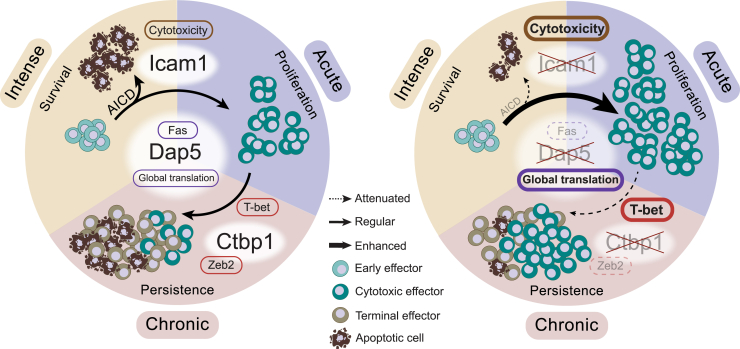
Unique and shared genes limiting T cell fitness identified in multimodal stimulation screens (Left) When effector T cells receive TCR stimulation, they undergo rapid proliferation accompanied by AICD, limiting expansion. When antigen-stimulation persists, cells eventually become terminally differentiated, apoptotic, or dysfunctional. (Right) Intense, acute, and chronic stimulation screens reveal factors regulating either common or specific T cell fitness traits. *Dap5* depletion in activated T cells stimulates global mRNA translation, upregulates cell cycle gene activity, and suppresses FAS expression, allowing cell pool expansion under all three stimulation conditions. *Icam1* ablation or Icam-LFA1 interaction blockade prevents T cell hyperclustering upon stimulation, allowing increased exposure to stimulation signals. This contributes to their stronger cytotoxicity and expansion, especially after intense and acute stimulation. On the contrary, *Ctbp1* depletion does not influence T cell expansion in the short run, but benefits their long-term persistence and functionality exclusively under chronic stimulation. It exerts this effect by hindering CTBP1/ZEB2/T-bet co-regulated effector terminal differentiation.

In this study, we uncovered and compared genes either exclusively, or commonly, contributing to T cell fitness under different modes of TCR stimulation. As the complex and dynamic nature of the TME has proven challenging to single out key factors, while maintaining high library coverage *in vivo*, we opted for a multimodal functional screen approach at genome-scale in defined settings. We performed three genome-wide CRISPR-Cas9 knockout functional screens in CD8 T cells upon different stimulations: intense, acute, and chronic, covering key aspects of effector biology, namely: survival, proliferation, and persistence. We identified several regulators previously reported by others, which not only confirmed their critical roles in controlling T cell antitumor efficacy, but extend those data by demonstrating their differential involvement in common, or specific, aspects determining T cell fitness. Furthermore, we uncovered, validated, and characterized several regulators not previously reported, which harness T cell fitness under either common (*Dap5* and *Icam1*), or exclusive (*Ctbp1*) T cell-stimulating conditions (Figure 6).

We identified *Dap5* as a critical negative regulator of T cell fitness under all three stimulation conditions. Its inactivation protects cells from cell apoptosis immediately following TCR stimulation, thereby increasing proliferation, cumulatively allowing for improved tumor control *in vivo*. DAP5 regulates mRNA translation,^130^^,^^131^^,^^132^^,^^133^^,^^134^^,^^135^ and its function can be influenced by stress, cell cycle, and apoptosis signaling pathways.^123^^,^^130^^,^^131^^,^^182^^,^^183^^,^^184^^,^^185^ *Dap5* ablation resulted in a global increase of translation, accompanied by a decline in 4E-BP1 protein. Polysome profiling indicated that the vast majority of mRNA was translated more efficiently. Cell cycle-regulating genes, such as *Ccnb1*, *Mki67*, *Ccne2*, and *Cenpe*,^186^^,^^187^^,^^188^^,^^189^ were induced in *Dap5*-KO T cells both pre- and post-stimulation, whereas activation-induced immunosuppressive genes, such as *Nr4a1*, *Pdcd1*, *Fas*, *and Tnfsf4*,^34^^,^^56^^,^^190^^,^^191^ were suppressed; suppression of both PD-1 and FAS was confirmed at the protein level. These observations may explain the phenotype induced by *Dap5* inactivation: an activated cell cycle program at baseline allows cells to achieve their effector status, resulting in attenuated activation and protection from AICD and dysfunction. Simultaneously, the increased capacity for global translation by *Dap5* ablation fuels the already activated and rapidly dividing cells. These traits are consistent with the observed fitness benefit upon all three stimulations. In combination with our TIL data, our results merit exploring the therapeutic benefit of lowering *Dap5* expression for T cell therapy.

Next, we focused on the group of genes involved in both intense and acute signaling; their perturbation protects cells from AICD while enhancing cell proliferation shortly after TCR stimulation, another feature of potential relevance for T cell therapies. The hits include multiple regulators of cell-cell interactions.^192^^,^^193^ Specifically, sgRNAs targeting *Icam1* and *Itgal* (encoding an LFA1 subunit), and *Fermt3* (integrin activator)^194^ were all highly enriched in the screens. Thus, interrupting cell-cell interactions is beneficial for the expansion of effector cells right after TCR stimulation, consistent with previous data.^147^ Mechanistically, perturbing ICAM1 surface expression leads to enhanced effector function. *Icam1-*KO effector cells exhibit stronger cytotoxicity, as judged by their transcriptional profile and functional readouts. This was not limited to mouse cells, as *ICAM1* and *ICAM3* co-depletion from human T cells produced a similar phenotype. These results suggest that disrupting ICAM1-mediated homotypic clustering enables T cells to proliferate more, while being less susceptible to undergo death. Our data predicts that pharmacologic interference with ICAM1/3 in human T cells may have translational value. This is supported by our clinical evidence showing that patients receiving TILs with low *ICAM1* expression have a better prognosis. As no ICAM3 antibody is currently available, we blocked the ICAM-LFA1 interaction using an LFA1 antibody, resulting in enhanced cell expansion and effector function. However, given the essential role of LFA1 in T cell extravasation from the endothelial compartment, its therapeutic targeting may be challenging.^195^^,^^196^ Notably, ICAM-LFA1-mediated clustering enables mutual costimulation, resulting in density-dependent self-regulation of proliferation and apoptosis.^148^ The inhibitory signal is likely dominated during CD3 stimulation, where rapid expansion and high-density culture happen. Therefore, blocking ICAM-LFA1 interaction may be beneficial for *ex vivo* T cell expansion where the inhibitory signal may exert a major influence.

Lastly, we identified and characterized genes contributing exclusively to chronic stimulation. Besides apoptosis resistance and enhanced proliferation, a growing body of evidence indicates that T cell persistence represents one of the key determinants of long-term immunotherapy responses.^76^^,^^77^^,^^78^^,^^197^^,^^198^ Several of our top hits were identified in previous screens (*Ccnc*,^92^ and *Regnase-1*^102^), are known to regulate T cell development (*Zeb2*,^157^ *Cd69*,^160^ and *Ets1*^199^), or were translated into a clinical target (*Cblb*^159^). Moreover, disrupting genes involved in mTORC1 regulation led to T cell expansion (*Lamtor4*, *Rraga*, and *Flcn*),^200^ in line with the finding that mTOR inhibition regulates stem-like CD8 cell development and exhaustion during chronic infection.^201^ We found that *Ctbp1* ablation in T cells enhanced persistence and effector function upon chronic tumor stimulation, both *in vitro* and *in vivo.* CTBP1 is a transcriptional regulator of a range of developmental processes and promotes cancer progression.^202^^,^^203^^,^^204^^,^^205^ However, its role in T cell differentiation is unknown. Our transcriptomic data suggest that upon chronic stimulation, *Ctbp1* inactivation pushes cells into a relatively active state accompanied by stalled terminal differentiation. Together with the IP-MS analysis, the results indicate a collaborative role of CTBP1, together with ZEB2, in regulating T-bet/ZEB2-induced effector terminal differentiation.^158^ This was supported by GSEA analysis, as well as *in vitro* and *in vivo* functional and phenotypic validation. Due to the oncogenic activity of *Ctbp1* in tumor development, its pharmacologic targeting may come with double benefit, increasing functional effector persistence for immunotherapy, while impacting on tumor cell growth.

In summary, we report an unbiased discovery of genes contributing either to individual or common fitness traits upon T cell stimulation. While confirming previously established regulators, we report several unknown genes and characterize their differential involvement in T cell fitness traits, specifically *Dap5*, *Icam1*, and *Ctbp1*. These screen hits merit preclinical exploration: whereas for some pharmacologic strategies may be developed (like antibodies for ICAM1-3, or small molecule inhibitors for DAP5 and CTBP1), we envisage also a shorter route to clinical translation, namely by genetic perturbation in T cell products for adoptive transfer, as is currently being explored for CAR T cells.^206^^,^^207^ Our comprehensive screens for different aspects of T cell fitness also provide the community with considerable functionally annotated gene lists for increasing our understanding of T cell stimulation. The computational interface we include with this manuscript may facilitate this exploration: https://rhpc.nki.nl/sites/hithub/app/.

## STAR★Methods

### Key resources table

**Table:** 

REAGENT or RESOURCE	SOURCE	IDENTIFIER
**Antibodies**		

Anti-human-CD50-APC	BioLegend	Cat# 330011, RRID: AB_1227570
Anti-human-CD102-PE	BioLegend	Cat# 328506, RRID: AB_2122200
Anti-human-CD54-BV421	BD Bioscience	Cat# 564077, RRID: AB_2738578
Anti-human-CD8-PB	BioLegend	Cat# 344717, RRID: AB_10551616
Anti-mouse-CCR7-BV605	BioLegend	Cat# 120125, RRID: AB_2715777
Anti-mouse-CD127-PE-Cy7	BioLegend	Cat# 121120, RRID: AB_2813991
Anti-mouse-CD137-APC	Miltenyi Biotec	Cat# 130-102-515, RRID: AB_2654997
Anti-mouse-CD25-BV605	BioLegend	Cat# 102035, RRID: AB_11126977
Anti-mouse-CD3-PE-Cy7	BioLegend	Cat# 100320, RRID: AB_312685
Anti-mouse-CD39-PE-Vio770	Miltenyi Biotec	Cat# 130-114-359, RRID: AB_2726585
Anti-mouse-CD44-AF488	BioLegend	Cat# 103015, RRID: AB_493678
Anti-mouse-CD54-BV421	BD Bioscience	Cat# 564704, RRID: AB_2738903
Anti-mouse-CD62L-APC	Miltenyi Biotec	Cat# 130-112-837, RRID: AB_2658860
Anti-mouse-CD62L-PE	Miltenyi Biotec	Cat# 130-112-836, RRID: AB_2658858
Anti-mouse-CD8-BV711	BioLegend	Cat# 100748, RRID: AB_2562100
Anti-mouse-CD95 (FAS)-PE-Vio770	Miltenyi Biotec	Cat# 130-120-291, RRID: AB_2801758
Anti-mouse-EOMES-AF647	BioLegend	Cat# 157703, RRID: AB_2814093
Anti-mouse-IFNγ-APC	Miltenyi Biotec	Cat# 130-123-283, RRID: AB_2819467
Anti-mouse-IL-2-FITC	Miltenyi Biotec	Cat# 130-110-179, RRID: AB_2652408
Anti-mouse-Ki67-PB	BioLegend	Cat# 652421, RRID: AB_2564489
Anti-mouse-KLRG1-PE-Cy7	BioLegend	Cat# 138416, RRID: AB_2561736
Anti-mouse-KLRG1-BV421	BioLegend	Cat# 138414, RRID: AB_2565613
Anti-mouse-LAG3-PE	Miltenyi Biotec	Cat# 130-111-513, RRID: AB_2656412
Anti-mouse-PD-1-PE	Miltenyi Biotec	Cat# 130-102-299, RRID: AB_2661364
Anti-mouse-PD-1-PerCP-Vio700	Miltenyi Biotec	Cat# 130-111-804, RRID: AB_2656938
Anti-mouse-T-bet-PE-Cy7	BioLegend	Cat# 644823, RRID: AB_2561760
Anti-mouse-TCR β chain-PE	BD Biosciences	Cat# 553172, RRID: AB_394684
Anti-mouse-TNF-PE	Miltenyi Biotec	Cat# 130-109-719, RRID: AB_2654209
Anti-mouse/human-Tox-PE	Miltenyi Biotec	Cat# 130-120-785, RRID: AB_2801785
Purified Rat Anti-Mouse CD16/CD32 (Mouse BD Fc Block™)	BD Biosciences	Cat# 553141, RRID: AB_394656
Anti-Eif4g2	Cell Signaling Technologies	Cat# 2182, RRID: AB_2095903
Anti-Tubulin, DM1A	Sigma Aldrich	Cat# T9026, RRID: AB_477593
Anti-4EBP1	Cell Signaling Technologies	Cat# 2855, RRID: AB_560835
Anti-mouse-HRP	Thermo Fisher Scientific	Cat# 62-6520, RRID: AB_2533947
Anti-rabbit-HRP	Thermo Fisher Scientific	Cat# G-21234, RRID: AB_2536530
Rabbit IgG Isotype Control	Thermo Fisher Scientific	Cat# 10500C, RRID: AB_2532981
Anti-CTBP1 antibody (D2D6)	Cell Signaling Technologies	Cat# 8684, RRID: AB_10859907
Anti-CtBP1 Clone 3/CtBP1 (RUO)	BD Biosciences	Cat# 612042, RRID: AB_399429
Anti-CD11a antibody	Bio X Cell	Cat# BE0192, RRID: AB_10948991
Anti-CD54	Bio X Cell	Cat# BE0020-2, RRID: AB_1107659
Isotype controls, mouse IgG1	Bio X Cell	Cat# BE0083, RRID: AB_1107784
Isotype controls, mouse IgG2a	Bio X Cell	Cat# BE0085, RRID: AB_1107771
Purified NA/LE Hamster Anti-Mouse CD178 Clone MFL3 (RUO)	BD Biosciences	Cat# 555290, RRID: AB_395708
Purified NA/LE Hamster IgG1 κ Isotype Control Clone A19-3 (RUO)	BD Biosciences	Cat# 553968, RRID: AB_395168
Anti-Mouse CD3e Functional Grade Purified	Thermo Fisher Scientific	Cat# 16-0031-86, RRID: AB_468849
Anti-Mouse CD28 Functional Grade Purified	Thermo Fisher Scientific	Cat# 16-0281-86, RRID: AB_468923
Anti-Human CD3 Functional Grade Purified	Thermo Fisher Scientific	Cat# 16-0037-85, RRID: AB_468855
Anti-Human CD28 Functional Grade Purified	Thermo Fisher Scientific	Cat# 16-0289-85, RRID: AB_468927

**Chemicals, peptides, and recombinant proteins**		

Mouse IL-2	ImmunoTools	12340026
Mouse IL-7	ImmunoTools	12340075
Mouse IL-15	ImmunoTools	12340155
Human IL-2	Novartis	Proleukin
Human IL-7	ImmunoTools	11340077
Human IL-15	ImmunoTools	11340157
LIVE/DEAD™ Fixable Near-IR Dead Cell Stain Kit	Thermo Fisher Scientific	L34976
CellTrace™ Violet Cell Proliferation Kit	Thermo Fisher Scientific	C34557
CellTrace™ Far Red Cell Proliferation Kit	Thermo Fisher Scientific	C34564
Sphero AccuCount, Blank particles, 5.26um, 10ml	Spehrotec	ACBP-50-10
OVA-tetramer-PE or BV421	In house^208^	NA
Retronectin	TAKARA	TB T100B
SureBeads Prot A 3mL	Biorad	1614013
HALT Protease and Phosphatase inhibitor cocktail	Fisher Scientific	78444
TrueGuide Synthetic tracrRNA, 20 nmol	Invitrogen	A35507
TrueCut™ Cas9 Protein v2	Invitrogen	A36499

**Critical commercial assays**		

BD™ Cytometric Bead Array (CBA) Human IFN-γ Flex Set	BD Biosciences	558269
BD™ Cytometric Bead Array (CBA) Human IL-2 Flex Set	BD Biosciences	558270
BD™ Cytometric Bead Array (CBA) Human TNF Flex Set	BD Biosciences	560112
BD™ Cytometric Bead Array (CBA) Mouse IFN-γ Flex Set	BD Biosciences	558296
BD™ Cytometric Bead Array (CBA) Mouse TNF Flex Set	BD Biosciences	558299
BD™ Cytometric Bead Array (CBA) Mouse IL-2 Flex Set	BD Biosciences	558297
NEBNext High Fidelity 2X PCR Master Mix	NEB	M0541L
Blood & Cell Culture DNA Maxi kit	Qiagen	13362
Isolate II RNA mini kit	Bioline	BIO-52072
Maxima First Strand cDNA kit	Thermo Fisher Scientific	15273796
Dynabeads CD8 positive isolation kit	Invitrogen	11333D
Dynabeads® Untouched™ Mouse CD8 Cells Kit	Invitrogen	11417D
P2 Primary Cell 4D-Nucleofector X Kit	Lonza	V4XP-2024
Click-iT™ HPG Alexa Fluor™ 594 Protein Synthesis Assay Kit	Thermo Fisher Scientific	C10429
Click-iT™ HPG Alexa Fluor™ 594 Protein Synthesis Assay Kit	Thermo Fisher Scientific	C10429
Annexin V Conjugates for Apoptosis Detection	Thermo Fisher Scientific	A13202
Annexin Binding Buffer (5X)	Thermo Fisher Scientific	V13246
Foxp3 / Transcription Factor Staining Buffer Set	eBioscience	00-5523-00

**Deposited data**		

CRISPR screens sgRNA library sequencing data	This paper	GSE251758
Dap5-KO vs Ctrl RNA and polysome sequencing data	This paper	GSE235709
Icam1-KO vs Ctrl RNA sequencing data	This paper	GSE235710
Ctbp1-KO vs Ctrl RNA sequencing data	This paper	GSE235707
Icam1-KO vs Ctrl proteomic data	This paper	PXD043545
Ctbp1 IP-MS data	This paper	PXD043545

**Experimental models: Cell lines**		

D4M.3A	Internal stock	RRID: CVCL_0P27
B16-F10	ATCC	RRID: CVCL_0159
MeVa2.1	Internal stock^209^	MeVa2.1
D10	Internal stock^210^	D10
A375	Internal stock	RRID: CVCL_0132
HEK293T	Internal stock	RRID: CVCL_0063
Platinum-E	Internal stock	RRID: CVCL_B488

**Experimental models: Organisms/strains**		

C57BL/6J mice	Janvier	000664; RRID: IMSR_JAX:000664
OT-1	The Jackson Laboratory	003831; RRID: IMSR_JAX:003831
Pmel-1	The Jackson Laboratory	005023; RRID: IMSR_JAX:005023
Rosa26LSL-Cas9 mice	The Jackson Laboratory	028551; RRID: IMSR_JAX:028551
OT-1/Cas9 mice	Bred in house	OT-1/Cas9 mice
Pmel-1/Cas9 mice	Bred in house	Pmel-1/Cas9 mice

**Oligonucleotides**		

Sequencing Primers	This paper	Table S6
sgRNA sequencing	This paper	Table S6

**Recombinant DNA**		

pMSCV-puro	Takara	634401
pCL-ECO	Addgene	RRID: Addgene_12371
Brie library	Addgene	RRID: Addgene_73633
lentiCRISPR v2	Addgene	RRID: Addgene_52961

**Software and algorithms**		

MAGeCK (v0.5.7)	(Li et al., 2014)^117^	https://sourceforge.net/projects/mageck/
REVIGO	(Supek et al., 2011)^211^	http://revigo.irb.hr/
GSEA (v4.1.0)	(Subramanian et al., 2005)^122^	http://www.gsea-msigdb.org/gsea/index.jsp
STAR (v2.6.0c)	(Dobin et al., 2013)^212^	https://github.com/alexdobin/STAR
FlowJo	BD Life Sciences	https://www.flowjo.com/
Graphpad Prism (v8.4.3)	GraphPad Software Inc.	https://www.graphpad.com/scientific-software/prism/
R	R Core Team	https://www.r-project.org/
HTSeq-count (0.11.4)	(Anders et al., 2014)^213^	https://pypi.org/project/HTSeq/
DESeq2 (1.36.0)	(Love et al., 2014)^214^	http://www.bioconductor.org/packages/release/bioc/html/DESeq2.html
MaxQuant (version 1.6.10.43)	(Cox et al., 2014)^215^	https://www.maxquant.org/
Perseus (version 1.6.10.43)	(Tyanova et al., 2016)^216^	https://maxquant.net/perseus/
STRING	(Szklarczyk et al., 2023)^138^	https://string-db.org/
Salmon	(Patro et al., 2017)^217^	https://combine-lab.github.io/salmon/
RiboDiff	(Zhong et al., 2017)^218^	https://public.bmi.inf.ethz.ch/user/zhongy/RiboDiff/
FeatureCounts	(Liao et al., 2014)^219^	https://subread.sourceforge.net/
LIMMA	(Law et al., 2014)^220^	https://bioconductor.org/packages/release/bioc/html/limma.html
TISCH2	(Sun et al., 2021)^126^	http://tisch.comp-genomics.org/
Seurat (v4.3.0)	(Hao et al., 2021)^221^	https://satijalab.org/seurat/index.html
CIPR	(Ekiz et al., 2020)^222^	https://github.com/atakanekiz/CIPR-Package

### Resource availability

#### Lead contact

Further information and requests for resources and reagents should be directed to and will be fulfilled by the lead contact, Daniel Peeper (d.peeper@nki.nl).

#### Materials availability

This study did not generate new unique reagents.

#### Data and code availability

CRISPR screen MAGeCK outputs are available as supplemental data. The raw sequencing data and the normalized read count from this study have been deposited in the Gene Expression Omnibus under the accession code, and are publicly available: GSE251758 (raw sequencing data and readcount for the screens), GSE235709 (*Dap5*-KO RNA and polysome sequencing), GSE235710 (*Icam1*-KO RNA-Seq), GSE235707 (*Ctbp1*-KO RNA-Seq). All mass spectrometry proteomic data generated in this study have been deposited to the ProteomeXchange Consortium via the PRIDE^223^ partner repository with the dataset identifier PXD043545. scRNA-Seq data for *CTBP1* expression in CD8 T cells of responders and non-responder was downloaded from the published dataset GSE120575.^181^ *CTBP1* expression in CD8 T cells and exhausted CD8 T cells from Pan-cancer patient cohorts was downloaded from the TISCH^126^ database (Table S2). Microscopy and western blot images reported in this paper will be shared by the lead contact upon request. This paper does not report original code. Any additional information required to reanalyze the data reported in this paper is available from the lead contact upon request.

### Experimental model and study participant details

#### Cell lines

Human D10,^210^ A375 (CVCL_0132), HEK293T (CVCL_0063) and platinum-E^224^ cell lines were retrieved from the Peeper laboratory cell line stock. The A375 melanoma cell line without endogenous HLA-A^∗^02:01 or MART-1 expression was transduced with lentiviral constructs encoding both components. The murine melanoma B16-F10 (CVCL_0159) cell line was obtained from ATCC, MeVa2.1^209^ and D4M (CVCL_0P27) cell lines were gifts from Dr. Christian Blank. The murine melanoma cell lines were lentivirus-transduced to express the full-length ovalbumin (OVA) protein. OVA-expressing cells were selected with hygromycin (250 μg/ml, 10687010, Life Technologies). All cell lines were cultured in DMEM (GIBCO), supplemented with 10% fetal bovine serum (FBS, Sigma) and 100 U/ml of Penicillin-Streptomycin (GIBCO). All cell lines were regularly tested for mycoplasma by PCR.^225^

#### Mouse model

For murine CD8 T cell isolation, to generate antigen specific Cas9-expressing mouse CD8 T cells, OT-I (The Jackson Laboratory) or Pmel-1 mice (The Jackson Laboratory) were crossed with Cas9-EGFP mice (C57BL/6 background, The Jackson Laboratory) and subsequently backcrossed for at least ten generations. Cas9-expressing OVA- or gp100-specific CD8 T cells were isolated from spleens of 8-20W male or female OT-I/Cas9 or Pmel-1/Cas9 mice, respectively. C57BL/6 (Janvier) mice or Cas9-EGFP mice (C57BL/6 background, either male or female, The Jackson Laboratory) were used as recipients for *in vivo* tumor models. All animal studies were approved by the animal ethics committee of the Netherlands Cancer Institute (NKI) and performed under approved NKI CCD (Centrale Commissie Dierproeven) projects according to the ethical and procedural guidelines established by the NKI and Dutch legislation. Mice were housed in single-use standard cages at controlled filtered air humidity (55%), temperature (21°C) and light cycle. All housing material, food and water were autoclaved or irradiated before use.

### Method details

#### Murine CD8 T cell isolation and *in vitro* cultures

Spleens from male or female OT-I/Cas9 mice were harvested and mechanically dissociated using a 100 μm and 70 μm cell strainer (Corning). The cell suspension was washed by centrifugation at 1000 xg using an isolation buffer (0.1% BSA in PBS). Red blood cells were lysed using a red blood cell lysis buffer (155 mM NH_4_Cl, 10 mM NaHCO_3_, 0.1 mM EDTA in distilled water; all Sigma) for five minutes. Cells were then washed once in PBS and once in isolation buffer and resuspended in isolation buffer. CD8 T cell isolation was performed using the Dynabeads Untouched Mouse CD8 Cells kit (11417D, Invitrogen) according to manufacturer’s instructions. Isolated naïve CD8 T cells were then resuspended in mouse CD8 T cell medium (RPMI, 10% FBS, 100 U/ml of Penicillin-Streptomycin, 10 ng/ml IL-2 (12340026, ImmunoTools), 0.5 ng/ml IL-7 (12340075, ImmunoTools), 1 ng/ml IL-15 (12340155, ImmunoTools) and 50 μM 2-mercaptoethanol (Merck)) at a concentration of 1x10^6^ cells/ml and primed using plate-bound CD3 antibody (0.25 μg/ 2x10^6^ cells, clone 145-2C11, Thermo Fisher Scientific) and CD28 antibody (2.5 μg/ 2x10^6^ cells, clone 37.51, Thermo Fisher Scientific) for 48 h. T cells were then either retrovirally transduced (see below) or maintained daily at a density of 1x10^6^ cells/ml for approximately 10 days before performing experiments (T cell stimulation by CD3 antibody or tumor-antigen).

#### Construction of retroviral vectors

To generate the retroviral sgRNA vector, the sgRNA cassette of the lentiCRISPR v2 (#52961, Addgene) was modified to replace the BsmbI sites with BbsI sites and subsequently cloned into the pMSCV puro backbone (634401, Clonetech) by restriction cloning. sgRNAs targeting genes of interest were either taken from the Brie library or generated using CHOPCHOP^226^ and cloned into the pMSCVpuro-sgRNA backbone by Golden-Gate cloning.^227^ The mAmetrine expressing retroviral sgRNA vector, pMSCVpuro-sgRNA-mAmetrine, was generated by inserting the mAmetrine fluorescent protein fragment after a mouse PGK promoter, upstream of the puromycin resistance sequence. The re-expression of wildtype or mutated ICAM1 in *Icam1*-KO cells was generated by inserting either a wildtype Icam1 fragment or an Icam1 fragment lacking the intracellular domain into a pMSCVpuro-sgRNA backbone after a mouse PGK promoter, upstream of the puromycin resistance sequence. For retroviral library construction, the sgRNA cassette of the Brie library (#73633, Addgene) was amplified by PCR and cloned into the pMSCVpuro backbone by restriction cloning. See Table S6 for oligonucleotide sequences used for sequencing and generating CRISPR-Cas9-mediated knockouts.

#### Retrovirus production and transduction of murine CD8 T cells

For retrovirus production, three million Platinum-E cells were seeded in a 10cm dish. After 24 h, these cells were transfected by polyethyleneimine (45 μg / 10 μg DNA, Polysciences) with 5 μg of pCL-ECO plasmid (#12371, Addgene) and 5 μg of the pMSCVpuro-sgRNA retroviral vectors. After another 24 h, the medium was replaced by Opti-MEM (Thermo Fisher Scientific) containing 2% FBS, 100 U/ml of Penicillin-Streptomycin. 24h later, the supernatant containing retrovirus was harvested, filtered through a 0.45 μm filter and stored at 4°C. Fresh medium was added to Platinum-E cells. The next day, supernatant was again harvested and filtered, combined with the supernatant of the first harvest and concentrated 10-20 times by spin-filter centrifugation (100 kDa pore size, Merck). The concentrated supernatant was snap frozen and stored at -80°C until used. For murine CD8 T cell transduction, 48 h CD3/CD28 antibody-primed T cells were harvested, one million primed OT-I/Cas9 T cells were mixed with 1 mL concentrated retroviral supernatant in a non-tissue culture treated 24-well plate pre-coated with Retronectin (25 μg/well, TB T100B, Takara). Cells were then spinfected at 3000 xg, 25°C for 1.5 h with minimum acceleration and no brake. After centrifugation, the plate was placed in the incubator. T cells were refreshed with mouse CD8 T cell medium 24 h after spinfection at the concentration of 1x10^6^ cells/ml medium. 48 h after spinfection, puromycin (4 μg/mL, Sigma) was added to the medium and cells were selected for at least 6d before starting experiments.

#### Genome-wide CRISPR screens (3 different settings) and MAGeCK analysis

Naive OT-I/Cas9 T cells were isolated, primed for 48 h using plate-bound CD3 antibody (0.25 μg/ 2x10^6^ cells, Thermo Fisher Scientific) and CD28 antibody (2.5 μg/ 2x10^6^ cells, Thermo Fisher Scientific) and transduced with the genome-wide Brie sgRNA library with at least a 1000x library representation. 1d after puromycin selection we harvested a library reference sample (t_0_). T cells were then cultured for an additional 5 days. 8 days post-transduction, we harvested a Pre-reactivation reference sample. The remainder of the transduced cell pool was resuspended in T cell stimulation medium (RPMI, 10% FBS, 100 U/ml of Penicillin-Streptomycin, 10 ng/ml IL-2 (12340026, ImmunoTools) and 50 μM 2-mercaptoethanol) at a cell density of 1x10^6^ cells/ml. Cells were then stimulated again under three different conditions: (1) Intense: selected CD8 T cells were stimulated with plate-bound CD3 antibody (1.25 ug/ 2x10^6^ cells) in non-tissue culture treated 24-well plates (CD3 stimulation plate). After 24 h, the same procedure was repeated by transferring cells to new CD3 stimulation plates. 24 h after the second-round of stimulation, dead cells were removed with the Dead Cell Removal kit according to manufacturer’s instructions (130-090-101, Miltenyi) and cells were harvested for analysis. (2) Acute: selected CD8 T cells were stimulated with CD3 antibody (1.25 ug/ 2x10^6^ cells) on 24-well stimulation plates for 24 h. Cells were then removed from the plates and refreshed daily at 1x10^6^ cells/ml with T cell stimulation medium for another 3 days before harvesting. (3) Chronic: for chronically stimulated samples, selected CD8 T cells were co-cultured with fresh D4M.OVA tumor cells in T cell stimulation medium for 11 days (11 times). Fresh tumor cells were added to T cells daily at a fixed T cell: tumor cell ratio. For the resting condition, cells were refreshed daily with T cell stimulation medium without adding tumor cells. Both stimulated and resting cells were harvested for analysis after 11 days (11 times) tumor stimulation. Final samples were collected by washing the T cells twice with PBS, after which the cell pellet was snap frozen and stored at -80°C until use. Genomic DNA was isolated using the Blood and Cell culture MAXI Kit (13362, Qiagen), according to manufacturer’s instructions. sgRNAs were amplified using a one-step barcoding PCR with NEBNext High Fidelity 2X PCR Master Mix (M0541L, NEB) and the forward and reverse primers (Table S6). The hexa-N nucleotide stretch contains a unique barcode identifying each sample following deep sequencing. MAGeCK (v0.5.7)^117^ was used to perform the analysis of the screen. To assess the depletion of core essential genes we compared the Pre-reactivation reference sample to the t_0_ library reference sample. We used shared core essential genes for all cell lines tested in the DepMap projects from the Broad and Sanger institutes as references.^111^^,^^228^^,^^229^ We filtered out from this list genes that were not expressed in our T cells (Table S1).

#### Isolation and generation of human MART-1-specific CD8 T cell

MART-1 TCR CD8 T cells were generated as previously described.^21^ In short, primary CD8 T cells were isolated from healthy male or female donor buffy coats (Sanquin, Amsterdam, the Netherlands), activated with plate-coated CD3 and CD28 antibodies (both 5 μg/ 2x10^6^ cells/ 24-well, 16-0037-85 and 16-0289-85, Thermo Fisher Scientific) for 48 h in primary human CD8 T cell medium (RPMI Medium (GIBCO) containing 10% human serum (H3667, Sigma-Aldrich), 100 U/ml of Penicillin-Streptomycin (GIBCO), 100 U/ml IL-2 (Proleukin, Novartis), 10 ng/ml IL-7 (11340077, ImmunoTools) and 10 ng/ml IL-15 (11340157, ImmunoTools)). Right after 48 h activation, T cells were removed from activation plates and spinfected with MART-1 TCR retrovirus on Retronectin-coated (25 μg/ 24-well, TB T100B, Takara) non-tissue culture-treated plates. Cells were harvested and maintained in primary human CD8 T cell medium 24 h after transduction. 1 week after transduction, MART-1 TCR expression was checked by flow cytometry (α-mouse TCR β chain, 553172, BD Pharmingen) and cells were cultured in human CD8 T cell medium (RPMI containing 10% fetal bovine serum (Sigma), 100 U/ml of Penicillin-Streptomycin (GIBCO) and 100 U/ ml IL-2 (Proleukin, Novartis)).

#### CRISPR-mediated knockout in human T cells

CRISPR-Cas9 knockout in activated human CD8 T cells was performed by nucleofection using the TrueGuide Synthetic gRNA system (Invitrogen) and the P2 Primary Cell 4D-Nucleofector X Kit S (V4XP-2024, Lonza). crRNA (Table S6, Invitrogen) and tracrRNA (A35507, Invitrogen) were annealed to a final duplex concentration of 20 μM and equimolar mixed with Cas9 (A36499, Invitrogen) to form ribonucleoproteins (RNP) prior to electroporation. T cells were resuspended in the transfection buffer (P2 Primary Cell 4D-Nucleofector X Kit S, V4XP-2024, Lonza) and mixed with RNPs. Electroporation was performed using the program EH-100 P2 (Lonza). Cells were maintained in human CD8 T cell medium, and were refreshed every 2 days. For *ICAM1/2/3*-KO efficiency check, 1 week after nucleofection cells were overnight stimulated with plate-bound CD3 antibody (1.25 ug/ 2x10^6^ cells), and sorted based on single or combined lack of expression of CD54 (ICAM1; clone HA58), CD102 (ICAM2; clone CBR-IC2/2) and CD50 (ICAM3; clone CBR-IC3/1) (all Bioscience). Sorted T cells were recovered for 1 week before experiments were performed. For *Dap5*- and *Ctbp1*-KO efficiency check, protein expression by Western blot analysis was applied.

#### *In vitro* T cell stimulation and viability assay

For short-term CD3 stimulation, activated murine CD8 T cells were either rested or stimulated with plate-bound CD3 antibody (1.25 ug/ 2x10^6^ cells) in T cell stimulation medium for 24 h for one (acute stimulation) or two rounds (intense stimulation). For short-term tumor-antigen-stimulation, activated T cells were challenged once with tumor cells expressing matching antigens in the T cell stimulation medium. For chronic CD3-stimulation, T cells were stimulated with plate-bound CD3 antibody (1.25 ug/ 2x10^6^ cells) in T cell stimulation medium. Cells were passed onto fresh coated plates every other day for 8 days. For extended chronic tumor-antigen-stimulation, matched fresh tumor cells were added to the T cell cultures every other day for a total of 3 weeks.

For short-term CD3-stimulation of human CD8 T cells, activated T cells were stimulated with plate-bound CD3 antibody (5 μg/ 2x10^6^ cells, 16-0037-85, Bioscience) for 24 h. For human CD8 T cells chronic tumor stimulation, activated MART-1 specific T cells were co-cultured with D10 melanoma cells every other day for at least 3 weeks.

T cell viability was analyzed after stimulation at the moment indicated in the figure legend. Viable cells were analyzed by CASY counter or flow cytometry according to the staining of DAPI or LIVE/DEAD™ Fixable Near-IR Dead Cell Stain Kit (L34976, Thermo Fisher Scientific). To obtain precise cell counts for flow cytometry analysis, Sphero AccuCount blank particles 5.26 μm (ACBP-50-10, Spherotec) were added to the samples. Data was processed using FlowJo software (BD Biosciences).

#### T cell-tumor co-culture cytotoxicity assay

Resting or stimulated mouse CD8 T cells were co-cultured with matching tumor cells at fixed T cell: tumor ratio. Days of co-culture depends on different T cells or tumor cell lines used in each experiment, as indicated in the figure legend. After co-culture, T cells were removed and remaining tumor cells were analyzed. T cell cytotoxicity was assessed by tumor colony formation in which the remaining tumor cells were fixed and stained for 1 h using crystal violet solution containing 0.1% crystal violet (CV, Sigma) and 50% methanol (Honeywell). For quantification, the remaining crystal violet was solubilized in 10% acetic acid (Sigma). Absorbance of this solution was measured on an Infinite 200 Pro spectrophotometer (Tecan) at 595 nm.

#### HPG translation assay

Ctrl and *Dap5*-KO T cells were used to assess the translational activity using the Click-iT™ HPG Alexa Fluor™ 594 Protein Synthesis Assay Kit (C10429, Thermo Fisher Scientific) according to manufacturer’s recommendations. In brief, 2x10^6^ CD8 T cells were stimulated with CD3 antibody for 24 h as described above. Cells were then collected and washed with 1 ml pre-warmed methionine-free DMEM and resuspended in 1 ml pre-warmed methionine-free DMEM containing 50 μM Click-iT® HPG. The samples were then incubated for 30 min at 37°C. Following the incubation time, cells were washed with PBS and transferred into a 96-well V-bottom plate. Samples were then permeabilized using the Fixation & Permeabilization Buffer Set (88-8824-00, Thermo Fisher Scientific). Cells were washed twice with 200 μl 3% BSA/PBS. 100 μl of Click-iT reaction cocktail was added per sample and incubated for 30 min at room temperature. The reaction cocktail was then removed and samples were washed with 100 μl Click-iT reaction rinse buffer and finally taken up in 200 μl FACS buffer for sample acquisition using flow cytometry.

#### Western blot

CD8 T cells were centrifuged at 1000 xg for 5 minutes and supernatant was removed. Cells were then washed with PBS twice before resuspending them in an appropriate volume of RIPA lysis buffer (50mM TRIS pH 8.0, 150mM NaCl, 1% Nonidet P40, 0.5% sodium deoxycholate, 0.1% SDS) supplemented with HALT Protease and Phosphatase inhibitor cocktail (78444, Fisher Scientific). Lysis was carried out on ice for 30 minutes. Samples were then centrifuged at 17000 xg and whole cell lysates were collected. Bio-Rad protein assay (500-0006, Bio-Rad) was used to quantify the protein content of each lysate. Protein concentrations were equalized and immunoblot samples were prepared by addition of 4xLDS sample buffer (15484379, Fisher Scientific) containing 10% b-Mercaptoethanol (final concentration 2.5%) and subsequent incubation of the samples at 95°C for five minutes. Proteins in lysates were size-separated on 4-12% Bis-Tris polyacrylamide-SDS gels (Invitrogen) and transferred to iBlot™ Transfer Stack (Invitrogen). Blots were blocked using 4% BSA in 0.2% Tween-20 in PBS. Blocked membranes were incubated with primary antibodies overnight. Immunoblots were developed using the Super Signal West Dura Extended Duration Substrate (34075, Thermo Fisher Scientific). The luminescence signal was captured by the Bio-Rad ChemiDoc imaging system. See key resources table for antibody list.

#### Flow cytometry

For surface protein staining, samples were collected and cells were spun down in V-bottom 96-well plates by centrifugation at 1000 xg for 5 min and washed twice with FACS buffer (0.1% BSA/PBS). Antibodies against surface markers of interest were diluted in the FACS buffer according to the manufacturer's instructions. Washed cells were then resuspended in 50 μl staining solution containing antibodies for 30 min on ice in dark. Following the staining step, cells were washed twice with FACS buffer by centrifugation at 1000 xg for 5 min. After washing, cells were resuspended in the FACS buffer for data acquisition. Dead cells were identified by positive DAPI (BD), or the LIVE/DEAD Fixable Near-IR (L34976, Thermo Fisher Scientific). For intracellular cytokine staining, samples were stimulated with 20 ng/ml PMA (Sigma) and 1 ug/ml Ionomycin (Sigma) for 4-5 h. 1 h after PMA/Ionomycin stimulation, GolgiPlug (555029, BD Bioscience) was added according to manufacturer's instructions to block the secretion of intracellular protein. Intracellular staining was performed with Foxp3/transcription factor staining buffer set after surface staining according to the manufacturer’s instructions (00-5523-00, eBioscience). Annexin V staining was conducted in combination with Annexin binding buffer (A13202, Thermo Fisher Scientific) according to manufacturer’s instructions. For surface and intracellular protein expression analysis, LSRFortessa Flow Cytometer or an LSR II Flow Cytometer (both BD) were used. Flow cytometry antibodies used in this study are listed in key resources table.

Secreted cytokine measurements in the cell culture supernatant of reactivated T cells were performed using the mouse IL2, TNF and IFNγ Cytometric Bead Array Flex set (558297, 558299, 558296, BD Biosciences) following manufacturer’s instructions. Flow cytometric analysis for CBA assay was performed using an iQue Screener PLUS (Intellicyte, Sartorius). All flow cytometric data was processed using FlowJo software (BD Biosciences).

#### Antibody blocking experiments

For antibody blocking experiments of CD8 T cells from healthy donor peripheral blood, T cell reactivation was performed as described above. T cell reactivation was performed in the presence of CD11a antibody (1 μg/ml, clone R7-1, BioXCell), CD54 (1 μg/ml, clone R6-5-D6, BioXCell) antibody, CD178/FasL (10 μg/ml, Clone MFL3 (RUO), BD Pharmingen) or respective isotype controls (1 μg/ml, mouse IgG2a, clone C1.18.4 and mouse IgG1, clone MOPC-21, both BioXCell; 10 μg/ml, Armenian Hamster IgG1, κ, BD Pharmingen). New antibodies were added during every medium refresh.

#### Immunoprecipitation mass spectrometry and sample preparation

Activated murine CD8 T cells were stimulated with CD3 antibody (1.25 ug/ 2x10^6^ cells) stimulation plates for 24 h. Cells were then harvested, washed twice with PBS and lysed in immunoprecipitation (IP) lysis buffer for 30 minutes. Two different IP lysis buffers were used for two independent experiments: Triton-X-100 IP buffer (30 mM Tris-HCl pH 7.4, 120 mM NaCl, 2 mM EDTA, 2 mM KCl, 1% Triton X-100) or NP40 IP buffer (50 mM Tris-HCl pH 7.4, 150 mM NaCl, 2 mM MgCl2, 0.1% NP-40), which both were supplemented with HALT Protease and Phosphatase inhibitor cocktail. The lysate was then centrifuged at 17000 xg for 10 minutes. Protein-containing supernatant was harvested and quantified. 8 mg of protein per sample was incubated with CTBP1 antibody (8684, CST) or isotype control (10500C, Invitrogen) and kept on a rotator for 2 h at 4°C. After incubation, pre-washed protein A beads (1614013, Bio-Rad) were added and incubated for another 2 h (Triton-X-100 IP buffer experiment) or overnight (NP40 IP buffer experiment). Beads were washed twice in the IP lysis buffer and once in PBS after immunoprecipitation. Washed beads were resuspended in 1x S-Trap lysis buffer and heated at 95°C for 7 min. in the presence of 20 mM DTT. Supernantants were transferred to new 1.5 mL tubes, after which proteins were alkylated with 40 mM iodoacetamide (30 min. at RT in the dark). Finally, proteins were digested o/n with 2 μg trypsin (Sigma-Aldrich) on S-Trap Micro spin columns according to the manufacturer’s instructions (ProtiFi, NY, USA). Peptides were eluted, vacuum dried and stored at -80°C until LC-MS/MS analysis. LC-MS/MS was performed using the same instrumentation and setup as described above, with the exception that a 90-min. gradient containing a 70-minute linear increase from 7% to 29% solvent B was applied for peptide separation. Immunoprecipitation mass spectrometry data were analyzed by MaxQuant (version 1.6.17.0) using standard settings with ‘match between runs’ selected. MS/MS data were searched against the Mus Musculus Swissprot database (17,042 entries, release 2020_07) complemented with a list of common contaminants. The maximum allowed mass tolerance was 4.5 ppm in the main search and 20 ppm for fragment ion masses. False discovery rates for peptide and protein identification were set to 1%. Trypsin/P was chosen as cleavage specificity allowing two missed cleavages. Carbamidomethylation on cysteines and methionine oxidation were set as fixed and variable modifications, respectively. LFQ intensities were log2-transformed in Perseus (version 1.6.14.0) (REF) after which protein abundance values were filtered for at least two valid values (out of 3) in at least one condition. Missing values were replaced by imputation based a normal distribution, using a width of 0.3 and a downshift of 1.8. Differentially expressed proteins were determined using a t-test (thresholds: p<0.05 and _2_Log LFQ abundance ratio < -1.0 ˆ > 1.0).

#### *In vivo* tumor growth experiment

0.5 x 10^6^ B16F10-OVA cells were subcutaneously (s.c.) injected in male or female C57BL/6 recipient mice. 4 d after tumor transplantation, 5 Gy total body irradiation (TBI) was applied to the mice. 1 day after TBI (day 5), 5 x 10^6^ sgCtrl or sg*Dap5*-expressing OT-I/Cas9 T cells were intravenously (i.v.) injected, 100.000 U hIL-2 (Proleukin, Novartis) were administered i.p. on day 5-7, and tumor growth was followed by measuring tumor volume three times weekly. Survival was measured according to tumor volume endpoint.

#### *In vivo* competition assay

sgCtrl and sg*Dap5*-expressing OT-I/Cas9 T cells were generated as described above. sgCtrl and sg*Dap5*-expressing T cells were stained with either CellTrace Violet (C34557, Thermo Fisher Scientific) or CellTrace Far Red (C34564, Thermo Fisher Scientific). Staining was performed according to manufacturer’s recommendations. A parallel experimental arm with swapped staining colors was included. sgCtrl and sg*Dap5* T cells were then mixed at a 1:1 ratio. 5 x10^6^ mixed T cells were transferred into B16F10-dOVA tumor-bearing male or female C57BL/6 mice 9 days after tumor transplantation. After 3 days, tumors and spleens were harvested. Samples were processed into single cell suspensions, stained for LIVE/DEAD and CD8 and analyzed by flow cytometry.

#### *In vivo* prolonged chronic tumor stimulation experiment

sgCtrl-mAmetrine and sg*Ctbp1*-mAmetrine-expressing OT-I/Cas9 T cells were generated as described above, using the pMSCVpuro-sgRNA-mAmetrine vector. 1 x 10^6^ 100 Gy irradiated (irr-) B16F10-OVA cells were first intraperitoneally (i.p.) injected to male or female Cas9-EGFP mice (C57BL/6 background), followed by i.v. injection of 5 x 10^6^ sgCtrl-mAmetrine or sg*Ctbp1*-mAmetrine-expressing OT-I/Cas9 T cells. 100.000 U hIL-2 (Proleukin, Novartis) i.p. injection was given twice per day in the first 3 consecutive days. Mice receiving T cells were challenged again with 1 x 10^6^ 100 Gy irr-B16F10-OVA 16d after the first irr-tumor challenge. On d22, 0.5 x 10^6^ healthy B16F10-OVA cells were s.c. injected to the mice. 7 days after healthy tumor injection, sentinel mice were sacrificed, transferred T cells isolated from tumors and spleens were stained and analyzed by flow cytometry. Tumor growth was followed by measuring tumor volume three times weekly, and survival was measured according to tumor volume endpoint.

#### T cell isolation from murine spleens, tumors and lymph nodes

Tumors were harvested and cut into small pieces, incubated in 5 mL tumor digestion medium (RPMI, 2% FBS, 10 U/mL DNAse I (Sigma), 200 U/mL collagenase type IV (Life Technologies)) at 37°C for 30 min while shaking. After digestion, tumor digests were then passed through 70 μm cell strainers (Corning), washed once with RPMI containing 10% FBS and once with PBS. Spleens were mechanically dissociated with syringes in RPMI containing 10% FBS and passed through 70 μm cell strainers (Corning). Samples were washed once with PBS and incubated in 2 ml red blood cell lysis buffer (155 mM NH_4_Cl, 10 mM NaHCO_3_, 0.1 mM EDTA in distilled water; all Sigma) for 5 min, followed by twice PBS wash. Lymph nodes were mashed in RPMI containing 10% FBS, and passed through 70 μm cell strainers (Corning), washed once with PBS. All samples were resuspended in the buffer used in downstream experiments.

#### Gene set enrichment analysis

GSEA analysis of screen results: Gene ontology term enrichment of biological process (GOBP) gene sets from each screen was performed by using GSEA software (v4.1.0)^121^^,^^122^ on the whole MAGecK output gene list, ranked by signed -Log_10_(MAGeCK Score). All gene sets that were either negatively or positively enriched (FDR <=0.25) in at least one screen were included. Go terms were then clustered using REVIGO.^211^ GO terms containing keywords or are related to “APOPTOSIS, CELL DEATH, T CELL, LYMPHOCYTE, ACTIVATION, PROLIFERATION and ADHESION” are included, while GO terms with irrelevant cell types (if mentioned in the name) were excluded. Heat map shows the -log_10_(FDR) value of each GO term enrichment.

For the analysis of CD8 T cell lineage gene sets enrichment from acute resolving and chronic viral infection models, all gene sets were derived from the scRNA-Seq data published in Nat Immunol. 2022.^107^ Top 50 differentially expressed genes in each cluster family (based on time frame and LCMV models) were used to generate gene sets used in this study: Acute_D8 (including CTL/EFF/MP clusters), Acute_D15 (including Trans I/Trans II/Trans CTL clusters), Acute_D30 (including Mem cluster), Chronic_>D15 (including Eff-like/prolif I/prolif II/pre-Exh clusters) and Chronic_>D15 (including Exh-Int/Exh-Prog/Exh-Term/ Exh-HSP/ Exh-Term Gzma/ Exh-KLR clusters). The enrichment of the five CD8 T cell lineage gene sets from each screen output was performed by using GSEA software (v4.1.0) as described above. And the 3 more relevant gene sets: Acute_D8, Acute_D15, Chronic_>D15 are shown.

For GSEA analysis on transcriptomic data of sgCtrl and sg*Ctbp1*-expressing OT-I/Cas9 cells after chronic stimulation, expression differences of the whole gene list ranked by stat values from the DESeq2 output was used as input. The Chronic_>D15 gene set was derived as described above. The ZEB1-KO_UP gene set (AIGNER_ZEB1_TARGETS) was taken directly from MSigDB database.^156^ The ZEB2-KO_UP gene set was derived from published database,^157^ genes with differential expression between ZEB2-deficient and -sufficient P14 CD8 T cells after LCMV infection are taken (29 upregulated genes). Gene sets “DAY8_EFFECTOR_VS_DAY30_EXHAUSTED_CD8_TCELL_LCMV_CLONE13_UP” (GSE41867),^73^ “KAECH_DAY8_EFF_VS_DAY15_EFF_CD8_TCELL_UP” and “KAECH_DAY8_EFF_VS_MEMORY_CD8_TCELL_UP” (GSE100001)^170^ were taken directly from MSigDB database as mentioned in the figure legend.

#### Proteomic analysis and sample preparation

For differential protein expression analysis of sg*Icam1* or sgCtrl-expressing OT-I/Cas9 cells, cells were stimulated with CD3 antibody for 24 h. Right after stimulation, cells were collected, washed twice with PBS and snap frozen. For protein digestion, frozen cell pellets were lysed in boiling Guanidine (GuHCl) lysis buffer.^230^ Protein concentration was quantified and diluted to 2 M GuHCl, and samples were digested twice (4 h and overnight) with trypsin (Sigma-Aldrich) at 37°C at an enzyme/substrate ratio 1:75. Digestion was quenched by the addition of TFA (final concentration 1%), after which the peptides were desalted on a Sep-Pak C18 cartridge (Waters). The eluates were vacuum dried and prior to mass spectrometry analysis, peptides were reconstituted again in 2% formic acid. Peptide mixtures were analyzed by nanoLC-MS/MS on an Q Exactive HF-X Hybrid Quadrupole-Orbitrap Mass Spectrometer equipped with an EASY-NLC 1200 system (Thermo Fisher Scientific). Samples were directly loaded onto the analytical column (ReproSil-Pur 120 C18-AQ, 1.9 μm, 75 μm × 500 mm, packed in-house) and eluted at a constant flow of 250 nl/min. Solvent A was 0.1% formic acid/water and solvent B was 0.1% formic acid/80% acetonitrile. For single-run proteome analysis, a 3 h gradient was employed containing a linear increase from 5% to 27% solvent B, followed by a 15-minute wash.

Proteome data were analyzed by MaxQuant (version 1.6.10.43)^215^ using standard settings. MS/MS data were searched against the Mus Musculus Swissprot database (17,027 entries, release 2020_02) complemented with a list of common contaminants. The maximum allowed mass tolerance was 4.5 ppm in the main search and 20 ppm for fragment ion masses. False discovery rates for peptide and protein identification were set to 1%. Trypsin/P was chosen as cleavage specificity allowing two missed cleavages. Carbamidomethylation was set as a fixed modification, while oxidation was used as variable modification. For Proteome data, LFQ intensities were log_2_-transformed in Perseus (version 1.6.10.43).^216^ Differentially expressed proteins were determined using t test (minimal threshold: FDR: 5% and S0: 0.1).

#### Protein-protein association and functional enrichment by STRING analysis

For the analysis of screen overlapping hits, 4 genes from the all-overlapping group (intense/acute/chronic) and 28 genes from the intense/acute overlapping group were taken as input for the STRING^138^ analysis for protein-protein interaction and functional enrichment analysis (32 genes in total). Genes involved in cell-cell interaction and extravasation are highlighted in the protein-protein association network. Enrichment of the top 15 GOBP gene sets (ranked by Strength, FDR< 0.01) was plotted.

For enrichment analysis of differentially expressed proteins between Ctrl and *Icam1*-KO OT-I/Cas9 T cells after 24 h CD3 antibody stimulation, proteins with significant (-log(p-value)>=1.3) difference in fold expression between reactivated *Icam1*-KO and control cells were used as input. Analysis was performed by STRING analysis with input either Up in *Icam1* or Down in *Icam1* (Up in WT) protein list. Gene sets with FDR<=0.1 were taken. And very small pathways (<15 genes) were excluded because of redundancy with larger pathways and too large pathways (>200 genes) were excluded since they are overly general. For comprehensive interpretation of enriched pathways, biological processes were only shown if the term “positive regulation of” or “negative regulation of” is mentioned.

#### Transcriptomic (RNAseq) analyses and sample preparation

For differential gene expression analysis between ctrl and gene-of-interest-KO T cells, cells were either stimulated or rested as described in the figure legend. To harvest the samples, CD8 T cells were sorted (for tumor stimulated samples) and pelleted, washed twice with PBS and resuspended in RLT buffer (Qiagen). The total RNA was isolated using the RNeasy Mini Kit (Qiagen), including an on-column DNase digestion (Qiagen), according to the manufacturer’s instructions. cDNA libraries were generated using the TruSeq Stranded mRNA sample preparation kit (Illumina Inc.) according to the manufacturer’s instructions and were sequenced on a HiSeq2500 or NextSeq 550 system (Illumina Inc.). Sequenced samples were mapped to the mouse genome (Mus.musculus.GRCm38) using STAR (v2.6.0c) in two-pass mode with default settings. Read counts were computed using HTSeq-count with default settings (0.11.4),^213^ normalization and statistical analysis of differential gene expression was performed using DESeq2 (v1.30.0). A sequencing batch effect is taken into account in the DESeq model by using the batch as a covariate.^214^

#### Polysome profiling analysis and sample preparation

Polysomal RNA isolation was performed as described previously.^231^ Briefly, Sucrose gradients for separation of polysomes were usually prepared by gentle sequential addition of 2.2 ml of the different sucrose solutions (e.i., 47, 37, 27, 17 and 7% in Tris-HCl pH 7.5 (20 mM), MgCl_2_ (10 mM) and KCl (100 mM), supplemented with 2 mM DTT (10197777001, Sigma), Ribosafe RNase inhibitor (1 μl/ml, BIO-65027, Bioline) and CHX (100 μg/ml, 239763, Sigma) into a 12 mL tube (Beckman, 9/16 × 3 1/2 in.) and left overnight at 4°C to achieve continuous gradient prior to the centrifugation. Cells were treated with 100 μg/mL CHX and harvest after washing with PBS with CHX and lysed. The lysates were centrifuged 1300 xg for 10 min at 4°C and the supernatants were transferred into new tubes. From the cleared lysates, 500 μL was loaded on top of each gradient, mounted on SW41TI rotor and centrifuged at 36000 rpm for 2 hr at 4°C. Following the centrifugation, each gradient was split into 15 equal fractions of 760 μl. Fractions 9-13 were collected for RNA isolation using TRIzol reagent (Thermo Fisher Scientific) according to the manufacturer’s instructions, polyA selected and followed by RNA library preparation as described above for mRNA-Seq.

#### Assessment of global translation efficiencies

We analyzed the generated RNA-Seq and polysome-Seq datasets in the following way. For both, initially, quality control was performed using the *FastQC* tool. Then, transcript quantifications were performed by Salmon,^217^ using the protein-coding transcript sequences from gencode vM21 annotation. All dataset-specific differential analyses (gene expression or polysome occupancy) were performed in an R environment, using the DESeq2 package.^214^ Differential translation efficiency analyses were performed using the RiboDiff tool,^218^ for which the input consisted of salmon-based transcript quantifications of primary transcripts that are determined based on Ensembl 96 APPRIS annotation. Genes with low sequencing depth were excluded from the translation efficiency analysis.

#### Survival analysis of patients receiving TIL therapy

The primary data are from a TIL trial conducted at the Sheba Medical Center (Trial number: NCT00287131 and NCT03166397, Tel Hashomer, Israel)^124^^,^^125^ and all patients (including both male and female) gave written informed consent. RNA was extracted from infused TIL products using Tri Reagent (#T9424, Sigma-Aldrich) according to the manufacturer’s protocol. RNA-Seq libraries were prepared with Illumina’s Ribo Zero Gold and TruSeq stranded library prep kits and sequenced on the Illumina HiSeq2500 platform using paired-end sequencing with read length of 2×125-150 bps. Reads were aligned to the human genome reference build hg38 using STAR aligner^212^ and were quantified with FeatureCounts.^219^ After filtration of lowly expressed genes (counts below 10 in more than 90% of samples), raw counts were normalized in the R environment according to the LIMMA pipeline.^220^ For survival analysis, we compared between the upper (top 33.3%) and lower thirds (bottom 33.3%) of patients, according to the expression of indicated genes. Kaplan-Meier plots were generated using the survival and survminer R packages. P-values for survival analysis were computed using the log-rank test.

#### Single cell analysis

For the Pan-cancer CD8 TILs expression of *DAP5, SERF2* and *CTBP1*, data was downloaded from TISCH2 database (Table S2).^126^ Studies containing both cell types of CD8 T cells (CD8T) and Exhausted CD8 T cells (CD8Tex) are included in the analysis (total 49 datasets). The expression level (log(TPM/10+1) of *DAP5, SERF2* and *CTBP1* in CD8T and CD8Tex cell types from each study were taken directly from the TISCH2 website, and the average expression was calculated.

For the analysis of *CTBP1* expression in CD8 T cells from responders and non-responders treated with ICB, the gene expression data and metadata information were downloaded from the TISCH2 database (GSE120575)^181^ and analyzed using Seurat (v4.3.0).^221^ scRNA-Seq for *CTBP1* expression in CD8 T cells in the context of responders and non-responders. Initially, metadata information (patient IDs and responses) was added to the gene expression data based on cell IDs. Cells with low read count (<200) were removed from the samples, followed by standard single cell analysis pipeline: normalization, scaling, dimension reduction with Uniform Manifold Approximation and Projection (UMAP) and clustering. The annotation of CD8 T cells from the sequencing data was performed by Cluster Identity Predictor (CIPR)^222^ based on the average expression of genes in the clusters. Identified CD8 T cells were analyzed for their *CTBP1* expression. Averages of *CTBP1* expression within responding and non-responding patient cohorts were compared.

### Quantification and statistical analysis

Details of the statistical analyses performed on each experiment are indicated in the respective figure legends. For biological experiments (non-omics), analyses were performed by Prism (Graphpad Software Inc., v8.4.3). Unless indicated, when comparing two groups, a Two-tailed Student’s t test was used for normally distributed data, and a two-tailed Mann-Whitney test was used for not normally distributed data. When comparing more than one group to the control group, one-way ANOVA with Holm-Sidak’s multiple comparisons test was performed when data is normally distributed, or Kruskal-Wallis test with Dunn’s post hoc test was used when data was not normally distributed. Tukey’s post-hoc analysis was used for multiple comparisons between all groups. Data distribution normality was analyzed by Shapiro-Wilk test. P value lower than 0.05 was considered as statistically significant. For *in vivo* experiments, sample size estimation for experimental study design was calculated by G^∗^Power.^232^

### Additional resources

Screen hits from this study can be visualized via the reader interface: https://rhpc.nki.nl/sites/hithub/app/

## Supplemental information

Document S1. Figures S1–S5

Table S1. Broad/Sanger common essential genes, screen MAGeCK output, GSEA analysis, overlapping hit list, related to Figures 1 and S1

Table S2. Gene expression (focused in this study) and survival of patients receiving TIL therapy, scRNA-seq analysis dataset list, *Dap5*-KO RNA-seq and polysome-seq data, related to Figure 2, S2, 3, 5 and S5

Table S3. Intense and acute stimulation screens overlapping hits STRING analysis, *Icam1*-KO RNA-seq and proteomic data, related to Figures 3 and S3

Table S4. *Ctbp1*-KO RNA-seq, IP-MS and GSEA data, T-bet/ZEB2 coregulatory gene list, related to Figure 4 and S4

Table S5. Gene lists for gene sets used in this study, related to Figures 1F, 4J and 4L

Table S6. All primers and sgRNA sequences used in this study, related to key resources table (Oligonucleotides) and STAR Methods (construction of retroviral vectors, genome-wide CRISPR screens (3 different settings) and MAGeCK analysis, CRISPR-mediated knockout in human T cells)

Document S2. Article plus supplemental information
