# Supplementary material for: Multimodal stimulation screens reveal unique and shared genes limiting T cell fitness
Source: Cancer Cell. 2024 Apr 8;42(4):623–645.e10. doi: 10.1016/j.ccell.2024.02.016 (PMC11003465; doi:10.1016/j.ccell.2024.02.016)
Supplement: Document S1. Figures S1–S5 [file mmc1.pdf]

Cancer Cell, Volume *42*

Supplemental information

Multimodal stimulation screens reveal unique and shared genes limiting T cell fitness

Chun-Pu Lin, Pierre L. Levy, Astrid Alflen, Georgi Apriamashvili, Maarten A. Ligtenberg, David W. Vredevoogd, Onno B. Bleijerveld, Ferhat Alkan, Yuval Malka, Liesbeth Hoekman, Ettai Markovits, Austin George, Joleen J.H. Traets, Oscar Krijgsman, Alex van Vliet, Joanna Poźniak, Carlos Ariel Pulido-Vicuña, Beaunelle de Bruijn, Susan E. van Hal-van Veen, Julia Boshuizen, Pim W. van der Helm, Judit Díaz-Gómez, Hamdy Warda, Leonie M. Behrens, Paula Mardesic, Bilal Dehni, Nils L. Visser, Jean-Christophe Marine, Gal Markel, William J. Faller, Maarten Altelaar, Reuven Agami, Michal J. Besser, and Daniel S. Peeper

Figure S1

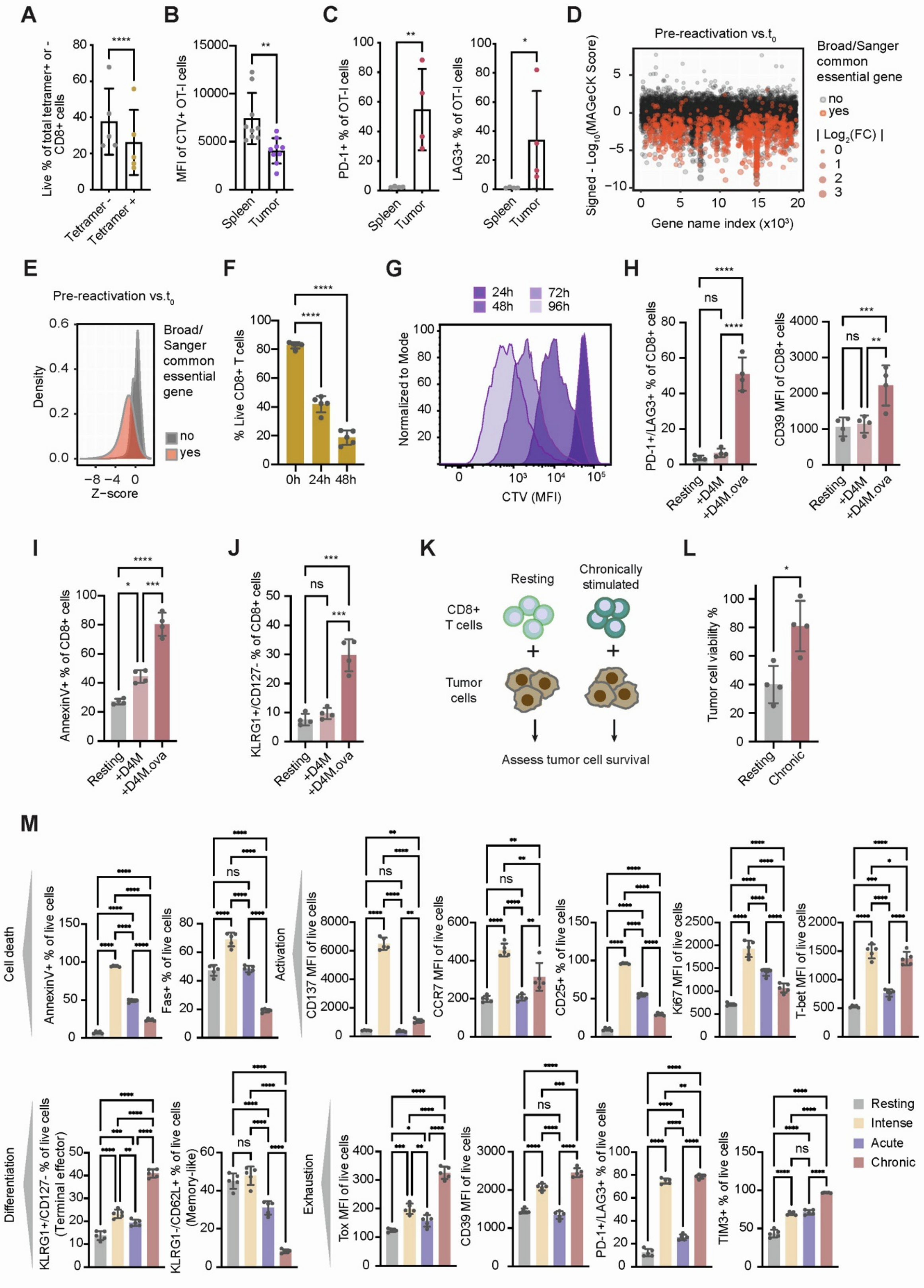
A
Live % of total tetramer+ or - CD8+ cells
Tetramer -
Tetramer +
B
MFI of CTV+ OT-I cells
Spleen
Tumor
C
PD-1+ % of OT-I cells
LAG3+ % of OT-I cells
D
Pre-reactivation vs.t0
Signed - Log10(MAGeCK Score)
Gene name index (x10³)
Broad/Sanger common essential gene
no
yes
| Log2(FC) |
E
Pre-reactivation vs.t0
Density
Z-score
Broad/ Sanger common essential gene
F
% Live CD8+ T cells
0h
24h
48h
G
24h
48h
72h
96h
Normalized to Mode
CTV (MFI)
H
PD-1+/LAG3+ % of CD8+ cells
CD39 MFI of CD8+ cells
Resting
+D4M
+D4M.ova
I
AnnexinV+ % of CD8+ cells
J
KLRG1+/CD127- % of CD8+ cells
K
Resting
Chronically stimulated
CD8+ T cells
Tumor cells
Assess tumor cell survival
L
Tumor cell viability %
Resting
Chronic
M
Cell death
AnnexinV+ % of live cells
Fas+ % of live cells
Activation
CD137 MFI of live cells
CCR7 MFI of live cells
CD25+ % of live cells
Ki67 MFI of live cells
T-bet MFI of live cells
Differentiation
KLRG1+/CD127- % of live cells (Terminal effector)
KLRG1-/CD62L+ % of live cells (Memory-like)
Exhaustion
Tox MFI of live cells
CD39 MFI of live cells
PD-1+/LAG3+ % of live cells
TIM3+ % of live cells
Resting
Intense
Acute
Chronic

Figure S1: Multimodal function-based genome-wide CRISPR knockout screens for genes contributing to T cell fitness upon differential stimulation, related to Figure 1

A) Percentage of live cells among SIINFEKL-tetramer-positive and -negative CD8 T cells from OVA^+ or OVA^- MeVa2.1 tumors. Statistical analysis was performed by two-tailed paired t-test (n=5 mice/ group).

B) Flow cytometry analysis of CellTrace Violet (CTV) staining on day 3 after transfer of OT-I/Cas9 T cells, isolated from B16.OVA tumor-bearing C57BL/6 mice. Statistical analysis was performed by two-tailed paired t-test (n=9 mice/ group).

C) Flow cytometry analysis of PD-1 and LAG3 surface expression on day 14 after transfer of OT-I/Cas9 T cells, isolated from B16.OVA tumor-bearing C57BL/6 mice. Statistical analysis was performed by two-tailed paired t-test (n=4 biological replicates).

D) Signed $-Log_{10}$(MAGeCK score) of gene inactivations comparing the t_0/library reference sample with the pre-reactivation sample. Highlighted are the Broad/Sanger common essential genes **(Table S1)**.[1]

E) Density plots of Z-scores of the Broad/Sanger common essential genes compared to non-essential genes when comparing the t_0/library reference sample to the pre-reactivation sample as in (D).

F) Viable T cell count after intensive CD3 stimulation. T cells were stimulated with CD3 antibody every 24h. Viable cells were analyzed by flow cytometry. Statistics were performed with one-way ANOVA, followed by a Dunnett post-hoc test (n=5 biological replicates).

G) Histograms depicting the CTV dilution of activated OT-I/Cas9 T cells at either 24, 48, 72 and 96h post CD3 stimulation.

H-J) Flow cytometry analysis of surface marker expression (H, J) and Annexin V staining (I) on OT-I/Cas9 T cells daily stimulated with D4M cell line with or without OVA expression for 12d. Resting cells were refreshed with medium only. Statistical analysis was performed with one-way ANOVA, followed by a Tukey post-hoc test (n=4 biological replicates).

K) Schematic outline of *in vitro* T cell-tumor co-culture killing assay.

L) Quantification of viable tumor cells from T cell-tumor co-culture killing assay. D4M.OVA tumor cells were co-cultured for 2d with OT-I/Cas9 T cells that had been either rested or chronically stimulated with D4M.OVA for 2wk. Viable tumor cells were

stained by CV and quantified by acetic acid solubilization. Resting, refresh medium only. Statistical analysis was performed by two-tailed paired t-test (n=4 biological replicates).

M) Flow cytometry analysis of marker expression from T cells stimulated with conditions performed in three different screens as in **Fig.1A**. Statistical analysis was performed by one-way ANOVA with Holm-Sidak's multiple comparisons test (n=5 biological replicates).

Error bars indicate SD. * P<0.05; ** P<0.01; *** P<0.001; **** P<0.0001.

Figure S2

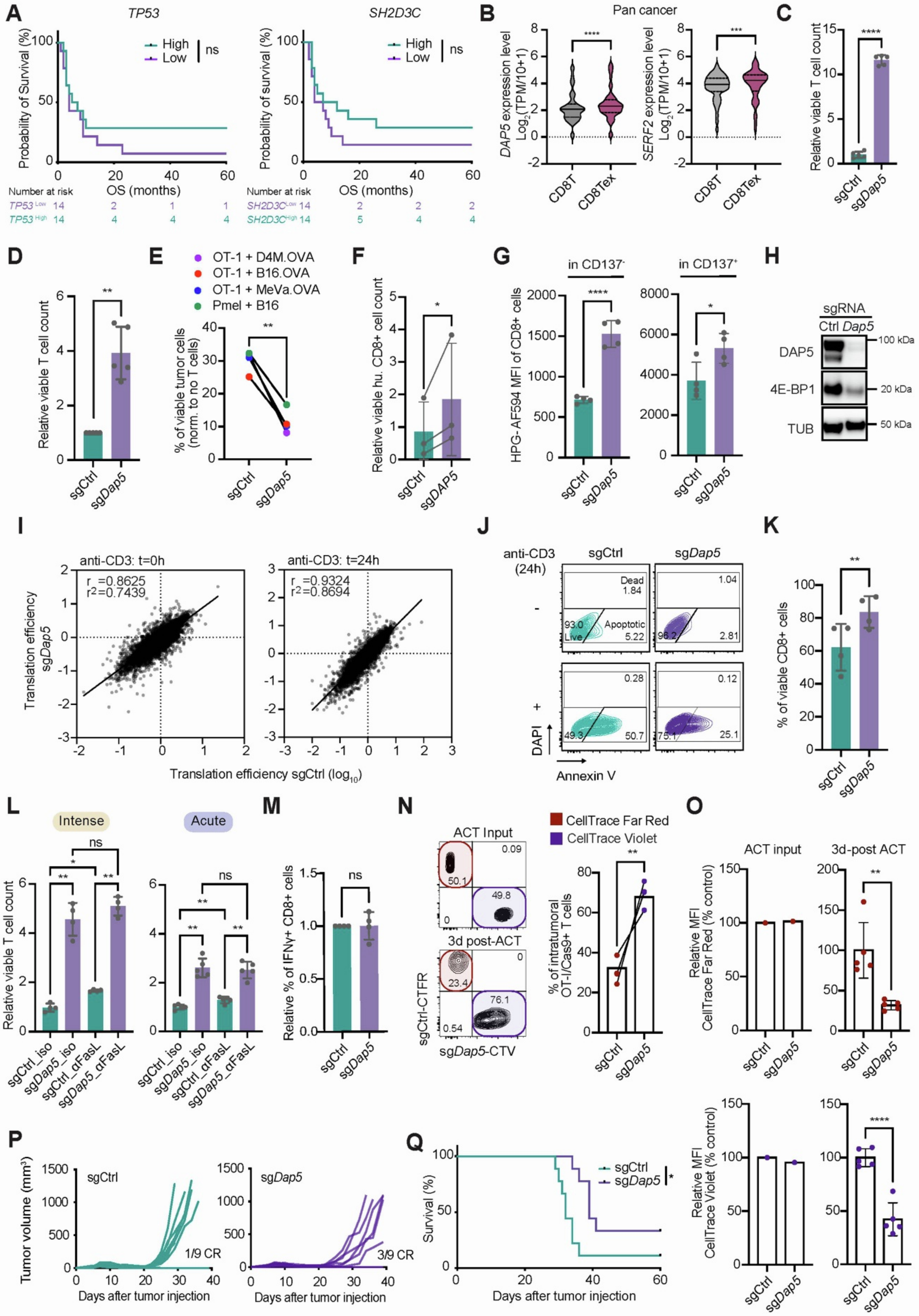
A
TP53
SH2D3C
High
Low
ns
Probability of Survival (%)
Probability of survival (%)
OS (months)
Number at risk
B
Pan cancer
DAP5 expression level Log2(TPM/10+1)
SERF2 expression level Log2(TPM/10+1)
CD8T
CD8Tex
C
Relative viable T cell count
sgCtrl
sgDap5
D
E
OT-1 + D4M.OVA
OT-1 + B16.OVA
OT-1 + MeVa.OVA
Pmel + B16
% of viable tumor cells (norm. to no T cells)
F
Relative viable hu. CD8+ cell count
sgDAP5
G
in CD137-
in CD137+
HPG- AF594 MFI of CD8+ cells
H
sgRNA
Ctrl Dap5
DAP5
4E-BP1
TUB
100 kDa
20 kDa
50 kDa
I
anti-CD3: t=0h
anti-CD3: t=24h
r =0.8625
r2=0.7439
r =0.9324
r2=0.8694
Translation efficiency sgDap5
Translation efficiency sgCtrl (log10)
J
anti-CD3 (24h)
Dead
Apoptotic
Live
DAPI
Annexin V
K
% of viable CD8+ cells
L
Intense
Acute
Relative viable T cell count
sgCtrl_iso
sgDap5_iso
sgCtrl_αFasL
sgDap5_αFasL
M
Relative % of IFNγ+ CD8+ cells
N
CellTrace Far Red
CellTrace Violet
ACT Input
3d post-ACT
sgCtrl-CTFR
sgDap5-CTV
% of intratumoral OT-I/Cas9+ T cells
O
ACT input
3d-post ACT
Relative MFI CellTrace Far Red (% control)
Relative MFI CellTrace Violet (% control)
P
Tumor volume (mm3)
1/9 CR
3/9 CR
Days after tumor injection
Q
Survival (%)
Days after tumor injection

Figure S2: *Dap5* inactivation alleviates global inhibition of effector T cell fitness and enhances tumor killing capacity, related to Figure 2

A) Kaplan-Meier OS curves of patients receiving TIL therapy (Besser cohort)[2,3] with top and bottom third highest and lowest (33.3%) *TP53* or *SH2D3C* expression in TIL products. Significance calculated by regular log-rank test.

B) Expression of *DAP5* and *SERF2* in CD8Tex and CD8T cells from 49 scRNA-Seq datasets (pan-cancer). Expression level was directly derived from TISCH2 website analysis **(Table S2)**.[4] TPM: Transcripts Per Million. Statistical analysis was performed by the Wilcoxon test from 49 independent datasets (n=49).

C) Quantification of viable OT-I/Cas9 T cells after chronic anti-CD3 stimulation. T cells expressing either sgCtrl or sg*Dap5* were repetitively stimulated with plate-coated CD3 antibody every other day for 8d. Statistical analysis was performed by two-tailed paired t-test. (n=5 biological replicates).

D) Quantification of surviving OT-I/Cas9 T cells, expressing either sgCtrl or sg*Dap5*, after co-culture with B16.OVA melanoma cells for 4d. Statistical analysis was performed by Mann–Whitney test (n=5 biological replicates).

E) Percentage of viable OVA- or gp100-expressing tumor cells after 3-4d co-cultured with either Ctrl or *Dap5*-KO OT-I/Cas9 or Pmel/Cas9 T cells, respectively. Percentage of viable tumor cells was calculated by normalizing to tumor only condition. Statistical analysis was performed by two-tailed paired t-test. Each data point represents the average tumor survival from 1-7 independent experiments of indicated matched tumor-T cell co-culture pairs.

F) Viable cell count of human *Dap5*-KO or Ctrl MART-1 T cells after CD3 antibody stimulation. T cells were stimulated with CD3 antibody on day 7 and day 14 after electroporation. Viable T cells were counted on day 21 post electroporation. Statistical analysis was performed by two-tailed paired t-test (n=3 biological replicates)

G) HPG incorporation in non-stimulated, $CD137^-$, or 24h CD3-stimulated $CD137^+$ Ctrl or *Dap5*-KO OT-I/Cas9 T cells. Statistical analysis was performed by two-tailed unpaired t-test (n=4 biological replicates).

H) Immunoblot showing the expression of DAP5, 4E-BP1 and Tubulin (loading control) in Ctrl or *Dap5*-KO OT-I/Cas9 T cells. Samples were collected from cells that were stimulated with CD3 antibody for 24h and subsequently cultured for an additional 72h.

I) Comparison of translation efficiencies between Ctrl or *Dap5*-KO T cells when unstimulated (left) or after 24h CD3 stimulation (right). Spearman correlation indicated in the upper left quadrant of each plot **(Table S2)**.

J) Representative (from n=2 biological replicates, other panel shown in **Figure 2K**) flow cytometry plots showing the assessment of T cell viability using nuclear DAPI and membrane AnnexinV staining. T cell viability was assessed from Ctrl or *Dap5*-KO T cells that were either not stimulated or stimulated with CD3 antibody for 24h.

K) Quantification of cell viability of Ctrl or *Dap5*-KO T cells right after CD3 24h-stimulation. Statistical analysis was performed by two-tailed paired t-test (n=4 independent experiments, each points represent mean of 3-4 biological replicates/ experiment)

L) Quantification of viable OT-I/Cas9 T cells. T cells expressing either sgCtrl or sg*Dap5* were stimulated by indicated condition in the presence of FasL blocking antibody or isotype. Statistical analysis was performed by one-way ANOVA with Holm-Sidak's multiple comparisons test (n=5 biological replicates).

M) Flow cytometry analysis of IFNγ intracellular staining in either Ctrl or *Dap5*-KO T cells, stimulated with CD3 antibody for 4-6h. Statistical analysis was performed by Mann–Whitney test (n=4-5 biological replicates).

N) Left: Flow cytometry plot showing T cell mixes either prior to *in vivo* transplantation or isolated from B16.OVA tumor 3d after ACT. Right: Quantification of *in vivo* competition assay. Labelling with either CTFR and CTV was swapped compared to the experiment depicted in **Fig. 2N** for robustness. Statistical analysis was performed by two-tailed paired t-test (n=5 mice/ group).

O) MFI of CTFR or CTV in Ctrl or *Dap5*-KO T cells, either prior to ACT or isolated from B16.OVA tumor 3d post ACT. Statistical analysis was performed by two-tailed unpaired t-test (n=5 mice/ group).

P) Individual tumor volume measurements of **Fig. 2O and 2P**. CR, complete responders.

Q) Kaplan-Meier plot depicting the survival of B16.OVA tumor-bearing mice treated with either Ctrl or *Dap5*-KO T cells. Significance was calculated with a regular log-rank test (n=9 mice/ group).

Error bars indicate SD. * P<0.05; ** P<0.01; *** P<0.001; **** P<0.0001.

Figure S3

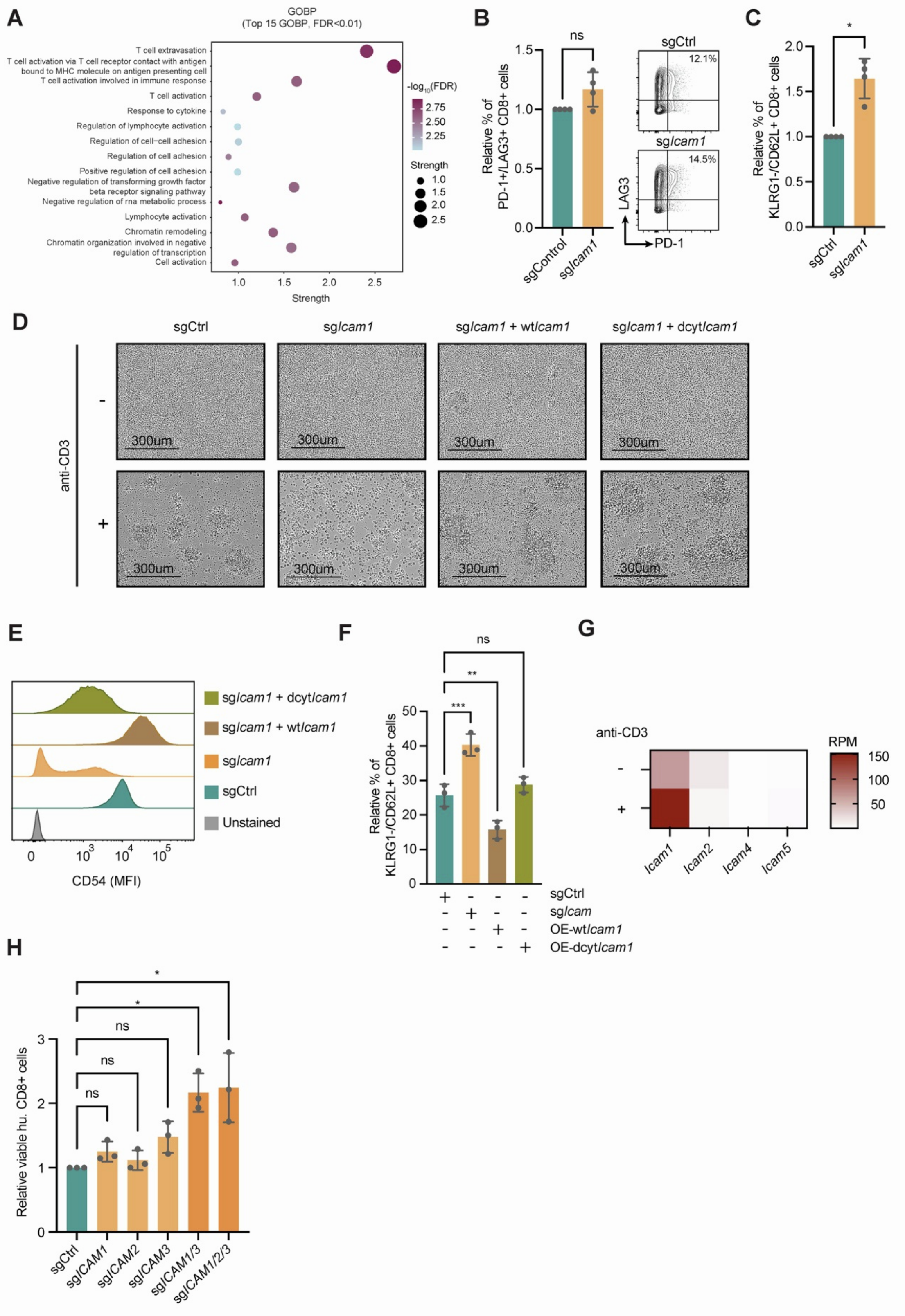
A
GOBP
(Top 15 GOBP, FDR<0.01)
T cell extravasation
T cell activation via T cell receptor contact with antigen bound to MHC molecule on antigen presenting cell
T cell activation involved in immune response
T cell activation
Response to cytokine
Regulation of lymphocyte activation
Regulation of cell-cell adhesion
Regulation of cell adhesion
Positive regulation of cell adhesion
Negative regulation of transforming growth factor beta receptor signaling pathway
Negative regulation of rna metabolic process
Lymphocyte activation
Chromatin remodeling
Chromatin organization involved in negative regulation of transcription
Cell activation
Strength
-log10(FDR)
B
Relative % of PD-1+/LAG3+ CD8+ cells
ns
sgControl
sgIcam1
sgCtrl
12.1%
sgIcam1
14.5%
LAG3
PD-1
C
Relative % of KLRG1-/CD62L+ CD8+ cells
sgCtrl
sgIcam1
D
sgCtrl
sgIcam1
sgIcam1 + wtIcam1
sgIcam1 + dcytIcam1
anti-CD3
300um
E
sgIcam1 + dcytIcam1
sgIcam1 + wtIcam1
sgIcam1
sgCtrl
Unstained
CD54 (MFI)
F
Relative % of KLRG1-/CD62L+ CD8+ cells
sgCtrl
sgIcam
OE-wtIcam1
OE-dcytIcam1
G
anti-CD3
RPM
Icam1
Icam2
Icam4
Icam5
H
Relative viable hu. CD8+ cells
sgCtrl
sgICAM1
sgICAM2
sgICAM3
sgICAM1/3
sgICAM1/2/3

Figure S3: Loss of *Icam1*-mediated homotypic T cell interactions amplifies CD8 T cell expansion and improves effector functions shortly after TCR stimulation, related to Figure 3

A) STRING enrichment analysis of shared targets from the top 50 enriched candidates of intense and acute stimulation screens (32 genes). Top 15 enriched GO biological process gene sets (ranked by enrichment strength) with FDR< 0.01 are shown **(Table S3)**.

B-C) Flow cytometry analysis of PD-1/LAG3 (B) and KLRG1/CD62L (C) surface expression on Ctrl or *Icam1*-KO T cells. T cells were stimulated with CD3 antibody for 24h and surface expression was assessed 1 week after stimulation. Statistical analysis was performed by Mann–Whitney test (n=4 biological replicates).

D) Images of morphological aberrations by microscopy for T cells expressing indicated sgRNA and overexpression constructs. Microscopy images were taken 48h after CD3 antibody stimulation (n=3 biological replicates). wt*Icam1*, wild type ICAM1; dcyt*Icam1*, ICAM1 lacking the intracellular domain.

E) Representative ICAM1 surface expression histograms of T cells as in (D). Cells were analyzed by flow cytometry 24h after CD3 antibody stimulation (n=3 biological replicates).

F) Flow cytometry analysis of KLRG1/CD62L surface expression of T cells as in (D). Cells were analyzed by flow cytometry 1wk after CD3 antibody stimulation. Statistical analysis was performed with one-way ANOVA with Holm-Sidak's multiple comparisons test (n=3 biological replicates).

G) mRNA expression of *Icam1-5* in murine CD8 T cells assessed by RNA-Seq before and after 24h CD3 stimulation. RPM, reads per million mapped reads.

H) Viable cell counts of Ctrl or *ICAM*-KO human T cells 1wk after CD3 stimulation. Human CD8 T cells were isolated from PBMC of healthy donors, *ICAM1/2/3* single, double or triple KOs were generated and cells were stimulated with CD3 antibody for 24h. Statistical analysis was performed by Friedman test with Dunn's post hoc test (n=3 biological replicates).

Error bars indicate SD. * P<0.05; ** P<0.01; *** P<0.001; **** P<0.0001.

Figure S4

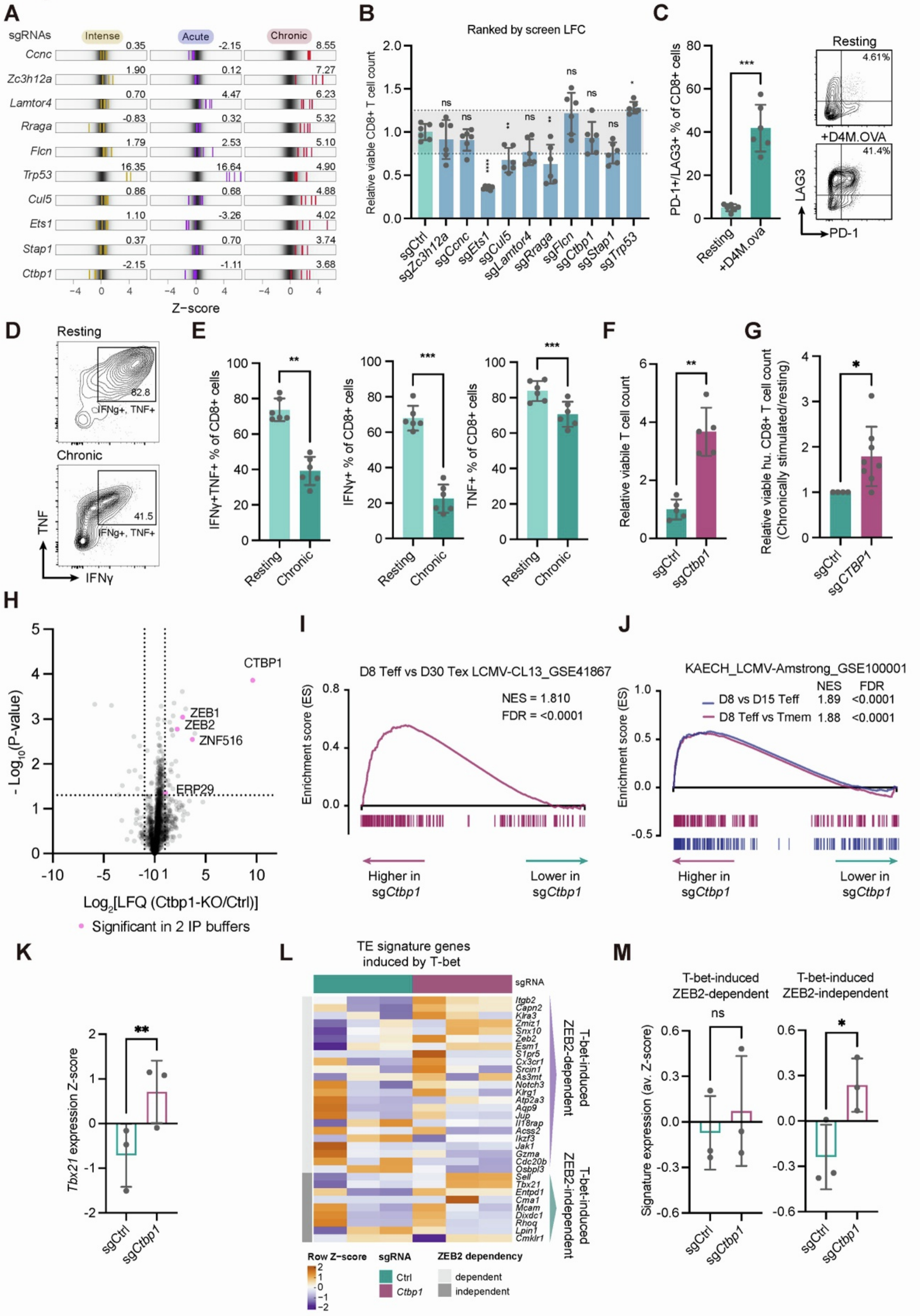
A
sgRNAs
Intense
Acute
Chronic
Ccnc
Zc3h12a
Lamtor4
Rraga
Flcn
Trp53
Cul5
Ets1
Stap1
Ctbp1
Z-score
B
Ranked by screen LFC
Relative viable CD8+ T cell count
C
PD-1+/LAG3+ % of CD8+ cells
Resting
+D4M.OVA
LAG3
PD-1
D
Resting
Chronic
IFNg+, TNF+
TNF
IFNγ
E
IFNγ+TNF+ % of CD8+ cells
IFNγ+ % of CD8+ cells
TNF+ % of CD8+ cells
F
Relative viable T cell count
G
Relative viable hu. CD8+ T cell count (Chronically stimulated/resting)
H
- Log10(P-value)
Log2[LFQ (Ctbp1-KO/Ctrl)]
Significant in 2 IP buffers
CTBP1
ZEB1
ZEB2
ZNF516
ERP29
I
D8 Teff vs D30 Tex LCMV-CL13_GSE41867
NES = 1.810
FDR = <0.0001
Enrichment score (ES)
Higher in sgCtbp1
Lower in sgCtbp1
J
KAECH_LCMV-Amstrong_GSE100001
D8 vs D15 Teff 1.89 <0.0001
D8 Teff vs Tmem 1.88 <0.0001
K
Tbx21 expression Z-score
L
TE signature genes induced by T-bet
T-bet-induced ZEB2-dependent
T-bet-induced ZEB2-independent
Row Z-score
sgRNA
ZEB2 dependency
dependent
independent
M
T-bet-induced ZEB2-dependent
T-bet-induced ZEB2-independent
Signature expression (av. Z-score)

Figure S4: *Ctbp1* ablation induces T cell persistence exclusively under chronic stimulation, associated with reduced ZEB2/T-bet-dependent terminal differentiation, related to Figure 4

A) Performance of top-ranking sgRNA hits from the chronic stimulation screen, across all three T cell stimulation screens. Numbers above plots indicate signed -Log_{10}(MAGeCK score).

B) *In vitro* validation of top-ranking hits from the chronic stimulation screen, showing relative viable T cell count after resting for 11d. Genes whose depletion caused ± 25% changes of viable cell count compared to Ctrl T cells under resting condition were excluded from further analysis. Statistical analysis was performed with one-way ANOVA, followed by a Dunnett post-hoc test from three biological replicates with two different sgRNAs per replicate (n=3x2). Dash lines: ± 25% difference in cell count compared to Ctrl cells.

C) Flow cytometry analysis for PD-1 and LAG3 surface co-expression of wt OT-I/Cas9 T cells that had been repetitively stimulated with or without D4M.OVA cells for 3wk. Resting: refreshing of medium only. Statistical analysis was performed by two-tailed paired t-test from three biological replicates with two different sgRNAs per replicate (n=3x2).

D) Representative flow cytometry plots for TNF^{+}/$IFN\gamma^{+}$ double positive population of wt T cells as in (C).

E) Quantification of D) and breakdown of TNF^{+} or $IFN\gamma^{+}$ single-positive populations. Statistical analysis was performed by two-tailed paired t-test from three biological replicates with two different sgRNAs per replicate (n=3x2).

F) Quantification of viable OT-I/Cas9 T cells after chronic anti-CD3 stimulation. T cells expressing either sgCtrl or sg*Ctbp1* were repetitively stimulated with plate-coated CD3 antibody every other day for 8d. Statistical analysis was performed by two-tailed paired t-test. (n=5 biological replicates).

G) Relative viable cell count of human *CTBP1*-KO or Ctrl MART-1 CD8 T cells after >3wk co-culture with D10 cells (expressing endogenous MART-1 antigen). Cell count fold change was normalized to resting condition. Statistical analysis was performed by Mann–Whitney test from four biological replicates with two different sgRNAs per replicate (n=4x2).

H) Immunoprecipitation mass spectrometry (IP-MS) analysis of CTBP1 from wt OT-I/Cas9 T cells upon CD3 stimulation (n=2 independent experiments with different IP

buffers, see also **Fig. 4K**) **(Table S4)**. Proteins identified from both CTBP1 IP-MS are highlighted in pink. 0.1% NP40 IP buffer was used for this experiment.

I) GSEA plot of the gene set "DAY8_EFFECTOR_VS_DAY30_EXHAUSTED_CD8_TCELL_LCMV_CLONE13_UP" (GSE41867), comparing *Ctbp1*-KO to Ctrl T cells after chronic tumor-antigen stimulation. GSEA was performed with all gene sets derived from LCMV-clone13 model from the source publication **(Table S4)**.[5]

J) GSEA plot of the gene set "KAECH_DAY8_EFF_VS_DAY15_EFF_CD8_TCELL_UP" and "KAECH_DAY8_EFF_VS_MEMORY_CD8_TCELL_UP" (GSE100001), comparing *Ctbp1*-KO to Ctrl T cells after chronic tumor-antigen stimulation. GSEA was performed with all gene sets from the source publication **(Table S4)**.[6]

K) Z-score of *Tbx21* (T-bet) expression from the transcriptomic data as in **Fig. 4I**. Statistical analysis was performed by two-tailed paired t-test (n=3 biological replicates).

L) Expression of terminally differentiated effector (TE) signature genes, which are known to be induced by T-bet but either dependent or independent of ZEB2 regulation,[7] in chronically tumor-antigen stimulated Ctrl or *Ctbp1*-KO T cells **(Table S4)**.

M) Quantification of (K). Statistical analysis was performed by two-tailed unpaired t-test (n=3 biological replicates).

Error bars indicate SD, unless otherwise specified. * P<0.05; ** P<0.01; *** P<0.001; **** P<0.0001.

Figure S5

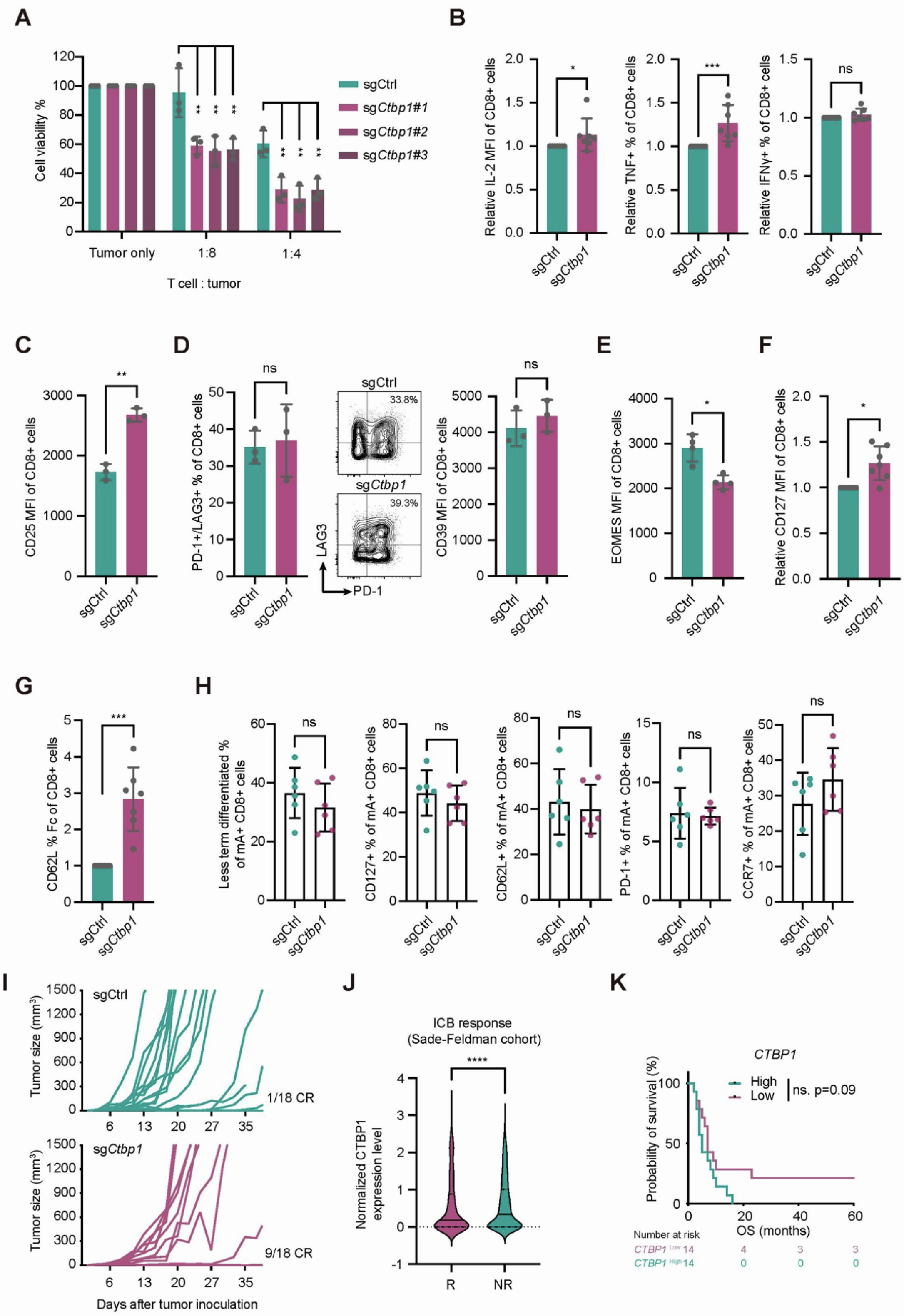
A
Cell viability %
sgCtrl
sgCtbp1#1
sgCtbp1#2
sgCtbp1#3
Tumor only
1:8
1:4
T cell : tumor
B
Relative IL-2 MFI of CD8+ cells
Relative TNF+ % of CD8+ cells
Relative IFNγ+ % of CD8+ cells
C
CD25 MFI of CD8+ cells
D
PD-1+/LAG3+ % of CD8+ cells
33.8%
39.3%
LAG3
PD-1
CD39 MFI of CD8+ cells
E
EOMES MFI of CD8+ cells
F
Relative CD127 MFI of CD8+ cells
G
CD62L % Fc of CD8+ cells
H
Less term differentiated % of mA+ CD8+ cells
CD127+ % of mA+ CD8+ cells
CD62L+ % of mA+ CD8+ cells
PD-1+ % of mA+ CD8+ cells
CCR7+ % of mA+ CD8+ cells
I
Tumor size (mm³)
1/18 CR
9/18 CR
Days after tumor inoculation
J
ICB response
(Sade-Feldman cohort)
Normalized CTBP1 expression level
R
NR
K
CTBP1
High
Low
ns. p=0.09
Probability of survival (%)
OS (months)
Number at risk
CTBP1 Low 14 4 3 3
CTBP1 High 14 0 0 0

Figure S5: Blocking CTBP1-mediated terminal T cell differentiation preserves T cell effector function and enables long-term tumor control, related to Figure 5

A) Quantification of CV staining from T cell-tumor co-culture killing assay. B16.OVA tumor cells were co-cultured with 3wk-chronically stimulated Ctrl or *Ctbp1*-KO OT-I/Cas9 T cells. After 4 days of co-culture, viable tumors were stained with CV and quantified by acetic acid solubilization. Statistics were performed with one-way ANOVA, followed by a Dunnett post-hoc test (n=3 biological replicates).

B) As in **Fig. 5B**, showing single cytokine positive populations.

C-G) As in **Fig. 5D-G**, showing surface (C-D, F-G) or intracellular (E) expression of the indicated protein. Statistical analysis was performed by two-tailed paired t-test (C-E) or Mann–Whitney test (F-G). Data points indicate biological replicates.

H) As in **Fig. 5I,** showing indicated protein surface expression on T cells isolated from lymph nodes. Less terminal differentiated population, KLRG1-/CD127+. Statistical analysis was performed by two-tailed unpaired t-test (n=6 biological replicates).

I) Individual tumor volume measurements from the *in vivo* experiment **(Fig. 5K)** illustrated in **Fig. 5H.** CR, complete responders.

J) *CTBP1* expression in CD8 T cells from responders (R) and non-responders (NR) to ICB.[8] Each data point indicates single CD8 T cells. In the violin plots, the center lines and dash lines denote the median and interquartile respectively. Statistical testing was performed by Mann-Whitney test.

K) Kaplan-Meier OS curves of patients receiving TIL therapy with high or low TIL *CTBP1* expression. Significance was calculated with a regular log-rank test.

Error bars indicate SD, unless otherwise specified. * P<0.05; ** P<0.01; *** P<0.001; **** P<0.0001.

REFERENCES

1. Dempster, J.M., Pacini, C., Pantel, S., Behan, F.M., Green, T., Krill-Burger, J., Beaver, C.M., Younger, S.T., Zhivich, V., Najgebauer, H., et al. (2019). Agreement between two large pan-cancer CRISPR-Cas9 gene dependency data sets. Nature Communications 2019 10:1 *10*, 1-14. 10.1038/s41467-019-13805-y.

2. Besser, M.J., Itzhaki, O., Ben-Betzalel, G., Zippel, D.B., Zikich, D., Kubi, A., Brezinger, K., Nissani, A., Levi, M., Zeltzer, L.a., et al. (2020). Comprehensive

single institute experience with melanoma TIL: Long term clinical results, toxicity profile, and prognostic factors of response. Molecular carcinogenesis *59*, 736-744. 10.1002/MC.23193.

3. Nissani, A., Lev-Ari, S., Meirson, T., Jacoby, E., Asher, N., Ben-Betzalel, G., Itzhaki, O., Shapira-Frommer, R., Schachter, J., Markel, G., and Besser, M.J. (2021). Comparison of non-myeloablative lymphodepleting preconditioning regimens in patients undergoing adoptive T cell therapy. Journal for ImmunoTherapy of Cancer *9*, e001743-e001743. 10.1136/JITC-2020-001743.
4. Sun, D., Wang, J., Han, Y., Dong, X., Ge, J., Zheng, R., Shi, X., Wang, B., Li, Z., Ren, P., et al. (2021). TISCH: a comprehensive web resource enabling interactive single-cell transcriptome visualization of tumor microenvironment. Nucleic Acids Research *49*, D1420-D1430. 10.1093/NAR/GKAA1020.
5. Doering, T.A., Crawford, A., Angelosanto, J.M., Paley, M.A., Ziegler, C.G., and Wherry, E.J. (2012). Network analysis reveals centrally connected genes and pathways involved in CD8+ T cell exhaustion versus memory. Immunity *37*, 1130-1144. 10.1016/J.IMMUNI.2012.08.021.
6. Kaech, S.M., Hemby, S., Kersh, E., and Ahmed, R. (2002). Molecular and functional profiling of memory CD8 T cell differentiation. Cell *111*, 837-851. 10.1016/S0092-8674(02)01139-X.
7. Dominguez, C.X., Amezquita, R.A., Guan, T., Marshall, H.D., Joshi, N.S., Kleinstein, S.H., and Kaech, S.M. (2015). The transcription factors ZEB2 and T-bet cooperate to program cytotoxic T cell terminal differentiation in response to LCMV viral infection. The Journal of experimental medicine *212*, 2041-2056. 10.1084/JEM.20150186.
8. Sade-Feldman, M., Yizhak, K., Bjorgaard, S.L., Ray, J.P., de Boer, C.G., Jenkins, R.W., Lieb, D.J., Chen, J.H., Frederick, D.T., Barzily-Rokni, M., et al. (2018). Defining T Cell States Associated with Response to Checkpoint Immunotherapy in Melanoma. Cell *175*, 998-1013.e1020. 10.1016/J.CELL.2018.10.038.
